# Genotypic performance of Australian durum under single and combined water-deficit and heat stress during reproduction

**DOI:** 10.1038/s41598-019-49871-x

**Published:** 2019-10-18

**Authors:** Haipei Liu, Amanda J. Able, Jason A. Able

**Affiliations:** School of Agriculture, Food & Wine, Waite Research Institute, The University of Adelaide, Urrbrae, SA Australia

**Keywords:** Plant stress responses, Abiotic

## Abstract

In Mediterranean environments, water deficiency and heat during reproduction severely limit cereal crop production. Our research investigated the effects of single and combined pre-anthesis water-deficit stress and post-anthesis heat stress in ten Australian durum genotypes, providing a systematic evaluation of stress response at the molecular, physiological, grain quality and yield level. We studied leaf physiological traits at different reproductive stages, evaluated the grain yield and quality, and the associations among them. We profiled the expression dynamics of two durum microRNAs and their protein-coding targets (auxin response factors and heat shock proteins) involved in stress adaptation. Chlorophyll content, stomatal conductance and leaf relative water content were mostly reduced under stress, however, subject to the time-point and genotype. The influence of stress on grain traits (e.g., protein content) also varied considerably among the genotypes. Significant positive correlations between the physiological traits and the yield components could be used to develop screening strategies for stress improvement in breeding. Different expression patterns of stress-responsive microRNAs and their targets in the most stress-tolerant and most stress-sensitive genotype provided some insight into the complex defense molecular networks in durum. Overall, genotypic performance observed indicates that different stress-coping strategies are deployed by varieties under various stresses.

## Introduction

Durum wheat (*Triticum turgidum* L. ssp. *durum*) is the only tetraploid wheat (2n = 4x = 28, genomes AABB) grown commercially worldwide, because of its unique grain characteristics and versatile end uses. Compared with bread wheat, durum is known for its grain hardness, high protein content, intense yellow pigmentation and unique nutty flavour^1^. Durum is mainly grown in Mediterranean environments such as the SEWANA region (South Europe, West Asia, and North Africa), where it is mostly rain-fed^2,3^. Soil conditions and environmental factors are important determinants of plant growth and development. In particular, drought and heat can severely limit the productivity and quality of staple crops like cereals, posing threats to global food security^4–7^. Due to reported climate change, the occurrence of soil water deficit and extreme temperatures are rapidly increasing^8,9^. Thus, the need to breed for cereal crops with higher yield stability and better stress adaptation is an issue of increasing urgency.

The key to achieving grain productivity in cereals is through successful reproductive development. Cereal reproduction largely depends on the flowering and grain filling processes, both of which are extremely sensitive to environmental conditions^10–12^. Water deficiency and heat stress can induce a series of physiological changes including photosynthetic efficiency, evapotranspiration, nutrient and water uptake, nutrient metabolism and transport^4,11,13,14^. Ultimately, these changes affect the reproductive structures, which not only limit the expression of yield potential, but also affect the quality of the harvested products, including starch content, protein concentration, bioactive compounds, vitamins and minerals^15–17^. However, the effects of stress on grain yield and quality may differ wildly due to different timing, severity and length of stress occurrences as well as whether a genotype is able to maintain plant fitness and adjust reproduction under that stress. Thus, germplasm should be evaluated in targeted environmental conditions to provide a better understanding of the basis of stress-adaptive traits^18^, which can then be used to help improve yield and stress tolerance.

In the Australian wheat belt, durum wheat is mainly grown in South Australia, New South Wales and western Victoria. Mild water-deficit stress usually occurs before flowering during spring and often continues to intensify through grain filling^13^. In wheat, water-deficit stress at pre-anthesis mostly affects floral initiation, pollen sterility and grain number^13,19,20^. Water deficiency that continues after anthesis when spikelet number and seed-set are mostly determined mainly affects the grain filling and grain size, through inhibition of dry matter accumulation^21^. Grain quality traits, such as starch content and protein accumulation, are also greatly affected by water deficiency after flowering, through its impacts on physiological processes such as photosynthesis and assimilate transport^21–23^. Our previous reports have investigated the morphological, physiological and yield traits under pre-anthesis water-deficit stress in a wide durum germplasm set, which exhibited significant genotypic differences. The reduction of grain yield ranged from 2.4% to 61.5% in the 20 genotypes studied^13^. Stress-tolerant and -sensitive Australian varieties differed in the physiological attributes of their leaves at early stages of stress suggesting a role for leaf responses in high yield stability^24^. Given that post-anthesis heat stress during reproductive stages is also quite common in Australian field conditions^15,25,26^, how these leaf physiological traits develop when pre-anthesis water deficit stress continues to grain fill and maturity, as well as when post post-anthesis heat stress occurs, is also of interest.

Studies of heat stress tolerance in cereals have been focused on two major types of heat stress^4,15,26^: heat shock and chronic heat. Extremely high temperatures (33–40 °C) for short periods of time (1–3 days) occurring periodically could be considered as heat shock while relatively mild heat stress (ranging from 20–32 °C) that lasts for a sustained period of time is considered as chronic heat stress^4,15,26,27^. Both stress conditions are known to cause significant yield penalty and impact on grain quality but with varied effects. In a pot experiment on durum and bread wheat, heat shock at 40 °C could reduce grain weight up to 96% when induced at an early reproductive stage (7 days after anthesis)^28^. However, although kernel protein content was greater than the control under different heat treatments, the response was variable ranging from 13.8% to 17.4% (compared to 13.2% in the control). In another study using bread wheat cultivars, both heat shock and chronic heat stress led to significant reduction in grain weight, with most-evident effects when day/night temperatures were higher than 30/25 °C^26^. A study in Chile on spring wheat showed that yield reduction under moderately high temperature (25–31 °C, maintained under heat shock regime) largely depended on the timing of the stress with the highest yield reduction rate (30%) recorded for heat stress at pre-anthesis^29^. This is also consistent with results obtained in South Australia where temperatures over 30 °C during anthesis resulted in almost double the yield loss during grain fill^27^. Given the conditions of the Australian wheat belt, short episodes of severe heat stress after flowering requires more research attention, as such conditions are predicted to be high-risk with rising incidence due to climate change^8,30,31^. Establishing with precision, how heat stress affects current Australian durum germplasm is therefore of significant interest to breeders so that they can make informed decisions through selection strategies.

Responses of crop plants are controlled by complex molecular processes activated upon stress perception. Recent advances in epigenetics suggest that genotypic diversity at the molecular level could be harnessed through epigenetic regulators, namely microRNAs (miRNAs), to provide new solutions for crop improvement^32,33^. miRNAs can precisely regulate the expression of their protein-coding target genes by inducing mRNA degradation or translational inhibition at the post-transcriptional level^32–36^. Compared with other mechanisms, miRNAs can rapidly respond to different developmental and environmental signals to modulate biological processes in plants. Under abiotic stress, miRNAs can reprogram the expression of downstream genes that tightly regulate adaptive physiological and/or reproductive traits, such as altered reproductive timing and alleviation of cellular damage^32,33,37^. Our previous work has examined the expression dynamics of durum miRNAs and their protein-coding targets under pre-anthesis water-deficit stress^24,38,39^. We have identified contrasting regulatory patterns of durum miR160 and two Auxin Response Factors (*ARF*s), *ARF8* and *ARF18*, at the early stages of stress in both stress-tolerant and -sensitive varieties^24,38^. *ARFs* play key roles in the cellular homeostasis and signalling of auxin, a plant hormone known to orchestrate a wide range of biological and physiological processes^40^. Thus miRNA-mediated molecular changes could contribute to the genotypic differences in the physiological traits, ultimately affecting yield components^24,32,38^. However, the expression dynamics of durum miR160 and the *ARFs* during later reproductive stages such as grain fill remains unknown. We have also identified a water-deficit stress-responsive miRNA, miR396, which specifically targets three genes that encode high-molecular weight heat shock proteins (HSP90)^24,38^. HSPs are chaperon proteins that contribute to protein thermostability, by assisting newly synthesized proteins to fold properly and existing proteins to stabilize at higher temperatures^41^. Whether the expression of miR396-*HSP*s changes in response to post-anthesis heat stress with or without the co-occurrence of water-deficit stress during grain development is therefore of interest. A recent study on two Italian durum cultivars with contrasting water-use efficiency has also revealed cultivar-specific expression profiles of durum miRNAs (including miR160 and miR396) in response to heat shock and drought stress at the vegetative stage^42^. These results further support that miRNA-mediated molecular responses are linked to cultivar performance under abiotic stress. Thus, a systematic expression analysis of the above-mentioned miRNAs at different time-points from grain fill until maturity, together with their targets involved in the stress response, is necessary. Furthermore, many reports have shown that the stress combination of water-deficit and heat often induces quite different molecular responses, which could not be extrapolated by observations under single stress conditions^43,44^. Analysis of the gene expression dynamics under single stress and stress combinations in parallel is therefore important to gain further information on the possible synergistic interactions between the two stress types.

Here, we present a systematic evaluation of selected Australian durum germplasm under single and combined pre-anthesis water-deficit and post-anthesis heat stress to enhance our understanding in this area. The main objectives were to: 1) investigate the responses of Australian durum germplasm to different types of stress at the molecular, physiological, yield and quality level; 2) identify high-performing germplasm under different stress conditions and genotypic variation in stress adaptation; and; 3) explore the possible associations among the studied traits for their potential in developing enhanced germplasm pools that can be used in breeding.

## Methods

### Plant materials and growing conditions

Four Australian durum varieties and six Durum Breeding Australia (DBA) University of Adelaide breeding lines were used in this study (Supplementary Table S1). The Australian durum varieties were selected based on their commercial value and their contrasting responses to pre-anthesis water-deficit stress^13,24^. The breeding lines are advanced selections from Durum Breeding Australia’s (DBA) southern-node breeding program. The performance of these ten genotypes under post-anthesis heat stress and combined stress was unknown. All seeds were provided by DBA.

Durum plants were grown in the South Australian Research and Development Institute (SARDI) glasshouse facility at the Waite Research Institute of the University of Adelaide. The standard growing conditions were 22 °C/12 °C (day/night) with a 12 h photoperiod. Seeds were germinated on filter paper and germinated young seedlings were transferred to pots (8.5 cm × 8.5 cm × 18 cm) containing 1.2 kg of N40 sand (a type of sandy soil commercially provided by the South Australian Research and Development Institute) and 0.5% CaCO_3_(one plant per pot). Basal nutrient solution was supplied as previously described^13^.

Four treatment groups were established for each genotype: control group (CG), pre-anthesis water-deficit stress group (WS), post-anthesis heat stress group (HS) and water-deficit plus heat stress group (WSHS). All treatment groups were well-watered to field capacity (12% soil water content) from germination to booting stage. From booting stage (Zadoks growth stage 43), water-deficit stress (soil water content at 6%, half of field capacity) was applied to WS and WSHS groups on a weight basis to simulate a rain-fed environment in South Australia as previously described^13^. All pots were monitored on a daily basis to ensure accurate maintenance of the soil water content and thus mitigate against any rapid fluctuations. Heat stress was applied by placing the pots in a growth chamber under 37 °C/27 °C (day/night) with a 12 h photoperiod. HS and WSHS plants were exposed to heat stress for 24 h at different stages of reproduction [5, 15, 25, 35, and 45 days post anthesis (DPA)] before plants were moved back to standard growing conditions between each treatment time-point. Anthesis date was defined as when 50% of anthers were extruded on the spike. CG plants were well-watered and remained under standard growing conditions until harvest. Supplementary Fig. S1 represents a schematic view of the stress treatment applications.

### Physiological responses, yield analysis and grain quality analysis

Chlorophyll content, leaf relative water content (RWC) and stomatal conductance (adaxial) were measured^13,24^ for each treatment group at 5, 15, 25, 35 and 45DPA. At harvest, yield components including grain weight per plant, number of grains per plant, biomass, plant height, and number of fertile tillers per plant were determined^13,45^. To compare the effects of different stress conditions on the genotypes, the reduction rate of each yield component of each genotype was calculated relative to their control. For example, the biomass reduction rate of genotype DBA Aurora under WS was calculated as (DBA Aurora WS biomass – DBA Aurora CG biomass)/DBA Aurora CG biomass × 100%. As described previously^13^, a rank summation index (Table 1 and Supplementary Table S2) was determined for genotype ranking purposes based on the yield component reduction rate under different types of stress. Genotypes with a higher rank summation index would have had less reduction in yield traits. Genotypes with a lower rank summation index had a higher reduction in yield traits. Statistical significance of all the reduction rates used for ranking is listed in Supplementary Table S3.

**Table 1:** Rank summation index of ten durum genotypes for their sensitivity to combined water-deficit and heat stress. Genotypes were ranked for each yield component and this then added together to derive the summation index (shown in bold).

Genotype	Biomass	Grain Weight	Harvest Index	Grain Number	1000-Grain Weight	Summation Index
DBA Aurora	10	10	8	9	10	**47**
L2	8	9	7	8	9	**41**
L1	6	7	10	7	8	**38**
WID802	9	8	4	10	2	**33**
L5	5	6	6	5	7	**29**
L4	7	5	3	6	5	**26**
DBA Spes	2	4	9	4	1	**20**
EGA Bellaroi	4	3	1	1	6	**15**
L3	3	2	2	3	4	**14**
L6	1	1	5	2	3	**12**

Genotypes with a higher rank summation index resulted in less yield loss and were more tolerant to stress. Genotypes with a lower rank summation index had more yield loss and were more sensitive to stress.

Wholemeal flour samples for measuring grain quality parameters were prepared using an IKA A11 analytical mill^45^. Protein content was determined using a Rapid N Elementar instrument based on the Dumas method (nitrogen content multiplied by the factor 5.7 at 11% moisture basis). Starch content was determined using the Total Starch Assay Kit (K-TSTA) from Megazyme according to the recommended procedure. Free, bound and total phenolic content of the wholemeal flour was determined as described previously^45^. Yellowness pigmentation (color b*, yellow-blue chromaticity) of the flour was measured using a Konica Minolta Chroma Meter^45^. Six biological replicates were used for leaf physiological traits and yield component measurements. Four biological replicates were used for grain quality measurements.

### Sampling, RNA extraction and qPCR

Stress-tolerant genotype DBA Aurora and stress-sensitive genotype L6 (based on the rank summation index in Table 1) were selected for gene expression analysis. Flag leaf samples from each treatment group were collected at 5, 15, 25, 35 and 45DPA with sterile razor blades and were snap-frozen in liquid nitrogen. Three biological replicates were collected for each treatment at each time-point. Total RNA was extracted using the Tri reagent (Sigma-Aldrich) and treated with TURBO DNase (ThermoFisher Scientific) following the manufacturer’s instructions. The concentration and quality of RNA samples were measured on a NanoDrop Lite spectrophotometer (ThermoFisher Scientific). RNA integrity was assessed by agarose gel electrophoresis and Bioanalyzer. cDNA was synthesized using the MystiCq microRNA cDNA Synthesis Mix Kit (Sigma-Aldrich) as previously described^24^. qPCR analysis of miR160a and the *ARF*s (*ARF8* and *ARF18*), miR396b and the *HSP90*s (CL1Contig1941, Contig102950, KukriC15_415)^24,38^ was performed using the PowerUp SYBR Green Master Mix (ThermoFisher Scientific) on a ViiA7 Real-Time PCR machine as previously described^24,38^. GAPDH was used as the reference gene. Primer sequences used in the study are included in Supplementary Table S4.

### Statistical analysis

Statistical analysis of all glasshouse data was performed as described previously^13,24^ using GENSTAT 15th Edn (VSN International Ltd). One-way ANOVA was performed to detect significant differences between four treatment groups for all physiological data (at each time-point), yield data and grain quality data, using the least significance difference (l.s.d.) at *P* < 0.05. For qPCR data analysis, the comparative CT (^ΔΔ^CT) method was used to calculate the relative expression of the miRNAs and their targets. One-way ANOVA was performed to detect significant difference between four treatment groups at each time-point within a genotype using the l.s.d. at *P* < 0.05. One-way ANOVA was performed to detect significant differences between time-points within each treatment group for all genotypes using the l.s.d. at *P* < 0.05 (Supplementary Table S5). The correlation coefficients between leaf physiological traits and major yield components were determined using GENSTAT 15th Edn (VSN International Ltd). Correlation of chlorophyll content and stomatal conductance with yield components was based on individual values of the replicates from each treatment group at each time-point per genotype (traits were measured on the same plants). Correlation of leaf relative water content with yield components was based on mean values of the replicates from each treatment group at each time-point per genotype.

## Results

### Genotype-dependent physiological responses to water-deficit and heat stress

**Figure 1:**
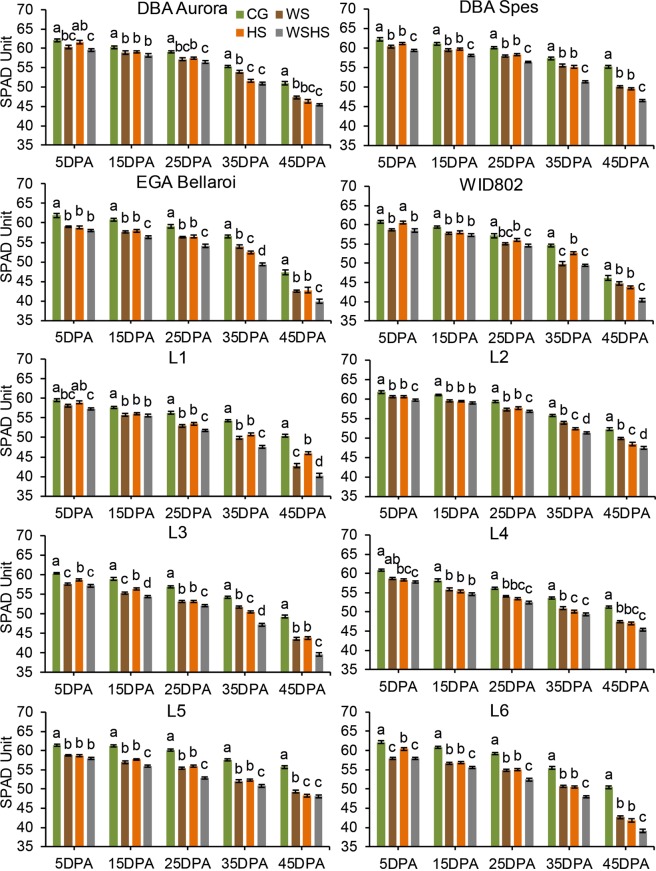
Chlorophyll content (SPAD units) of ten durum wheat genotypes at five time-points in four treatment groups. Means ± SE for *n* = 6 are shown. Different letters (a–d) denote statistically significant differences at the *P* < 0.05 level among treatment groups within a time-point. DPA, days post anthesis. CG, control group; WS, water stress group; HS, heat stress group; WSHS, combined water stress and heat stress group.

Under WS, the chlorophyll content was significantly decreased compared with CG at all five time-points for all ten genotypes (except for L4 at 5DPA) (Fig. 1). Under HS, chlorophyll content was significantly decreased from 15DPA to 45DPA for all genotypes. Under WSHS, a significant decrease compared with CG was observed for all genotypes at all time-points. When comparing the chlorophyll content between two single stress groups, genotype-dependent trends were observed. For example, there was no significant difference between WS and HS at all time-points for DBA Spes. Chlorophyll content under HS was significantly higher than under WS for L3 at 5DPA and 15DPA. When comparing the single stress groups with the combined stress, the trends were also genotype-dependent. For example, in DBA Spes, WSHS was constantly lower than both WS and HS at all time-points. For WID802, there was no difference between WS and WSHS until the last time-point. Time series analysis revealed developmental changes of chlorophyll content within each treatment group (Supplementary Table S5). As an example, under CG and HS, EGA Bellaroi started exhibiting a significant decline in chlorophyll content at 25DPA compared with 5DPA (*P < *0.001). However, under WS and WSHS, a significant drop was found early at 15DPA (*P < *0.001, compared with 5DPA), suggesting that water deficiency impacted chlorophyll content sooner than HS alone.

**Figure 2:**
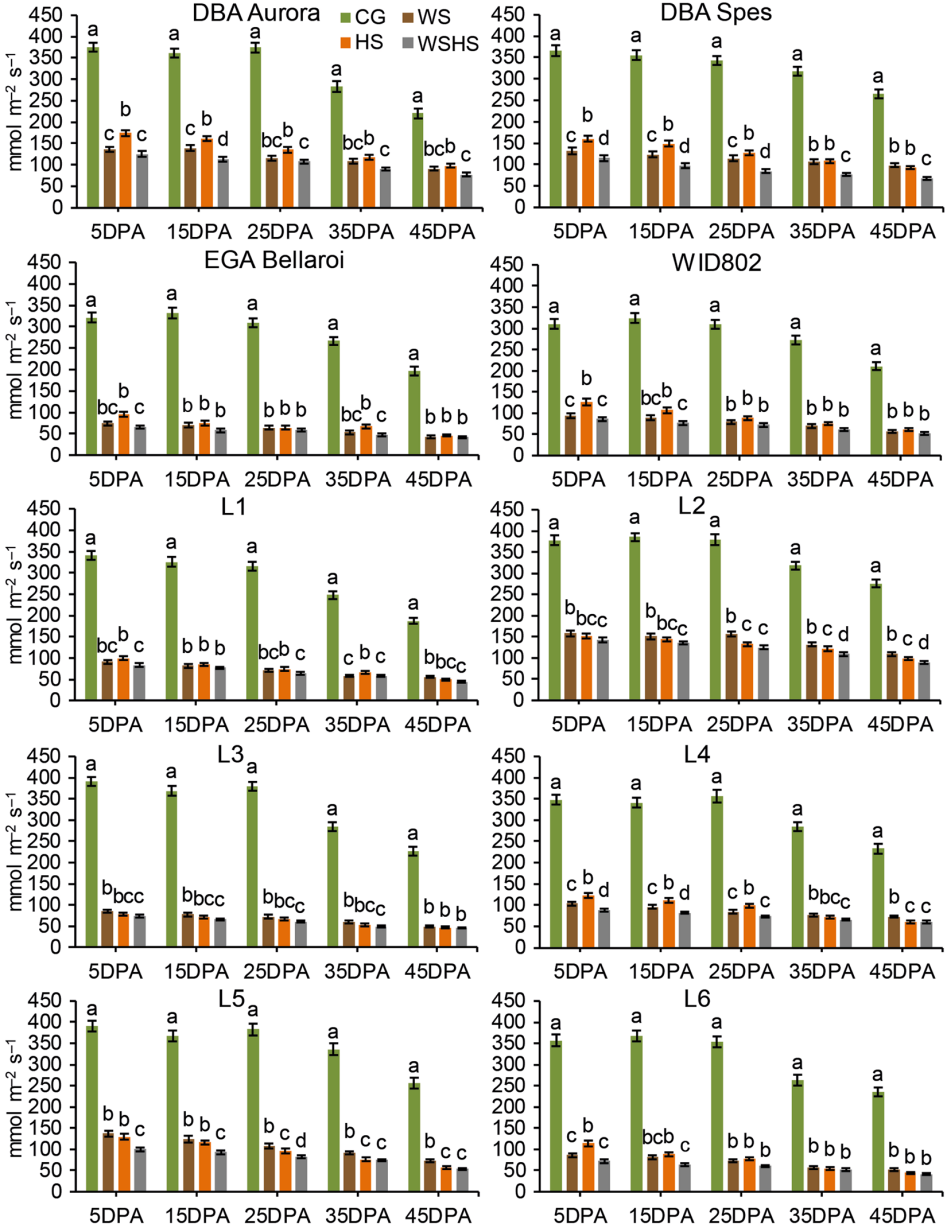
Stomatal conductance (adaxial) of ten durum wheat genotypes at five time-points in four treatment groups. Means ± SE for *n* = 6 are shown. Different letters (a–d) denote statistically significant differences at the *P* < 0.05 level among treatment groups within a time-point. DPA, days post anthesis. CG, control group; WS, water stress group; HS, heat stress group; WSHS, combined water stress and heat stress group.

For stomatal conductance, all three stress groups exhibited significant reduction for all genotypes at all time-points (Fig. 2). Similar to chlorophyll content, genotype-dependent responses were found when comparing the single stress groups and the combined stress group. For DBA Spes, WSHS had significantly lower stomatal conductance than both WS and HS at all time-points. DBA Aurora had significantly lower stomatal conductance in WSHS than HS at all time-points. Interestingly, WID802 and L6 exhibited the exact same pattern in terms of difference between the groups – no difference was found for stomatal conductance between WS, HS and WSHS after 25DPA. Genotypic differences were also found for the time series analysis on stomatal conductance (Supplementary Table S5). For example, under the control condition, both DBA Aurora and L3 did not have significant changes in stomatal conductance until 35DPA (compared with 5DPA, *P < *0.001). However, under WSHS, stomatal conductance in L3 significantly declined at 15 DPA while for DBA Aurora it commenced at 25DPA. The results suggest that the impact of WSHS on stomatal conductance develops differently across different genotypes.

**Figure 3:**
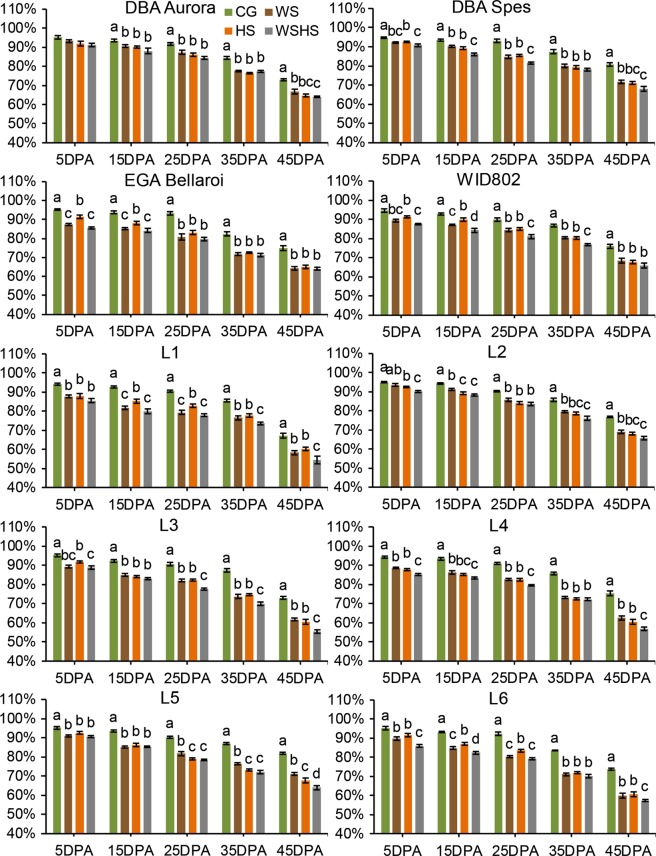
Leaf relative water content of ten durum wheat genotypes at five time-points in four treatment groups. Means ± SE for *n* = 6 are shown. Different letters (a–d) denote statistically significant differences at the *P* < 0.05 level among treatment groups within a time-point. When no letters were shown there was no statistical difference (*P* > 0.05) detected among the treatment groups. DPA, days post anthesis. CG, control group; WS, water stress group; HS, heat stress group; WSHS, combined water stress and heat stress group.

For leaf RWC, all three stress groups exhibited a significant reduction for all genotypes at all time-points except for DBA Aurora and L2 at 5DPA (Fig. 3). When comparing the RWC between WS and HS, genotype-dependent trends were observed. For DBA Aurora, DBA Spes, L3 and L4, there was no significant difference between WS and HS at all time-points. However, for EGA Bellaroi, HS had significantly higher RWC than WS at 5DPA and 15DPA. Significantly lower RWC in WSHS than single stress groups tended to occur in later reproductive stages. For example, the RWC of WSHS was significantly lower than both WS and HS starting from 25DPA in L3, and from 35DPA in L1. Similarly, the time series analysis of RWC exhibited different patterns dependent on the genotype (Supplementary Table S5). For example, for L2, both CG and WSHS did not exhibit significant changes in RWC until 25DPA (*P* < 0.001). However, for L6, the RWC started to drop significantly in the CG and WSHS at 15DPA (*P* < 0.001).

Phenotypic differences could also be observed across the ten genotypes under the stress treatments. Some genotypes exhibited severe stress-induced symptoms while other genotypes managed to grow and remain relatively healthy. For example, in stress-sensitive genotype L6, plants under WS, HS and WSHS showed significantly reduced leaf greenness, reduced biomass and aborted tillers when compared with the control (Supplementary Fig. S2). However, for stress-tolerant genotype DBA Aurora, the plants in the stressed groups appeared similar in growth to the control at early time-points, and with less severe symptoms than L6 in later time-points (Supplementary Fig. S3).

### Effects of water-deficit and heat stress on the yield components

**Figure 4:**
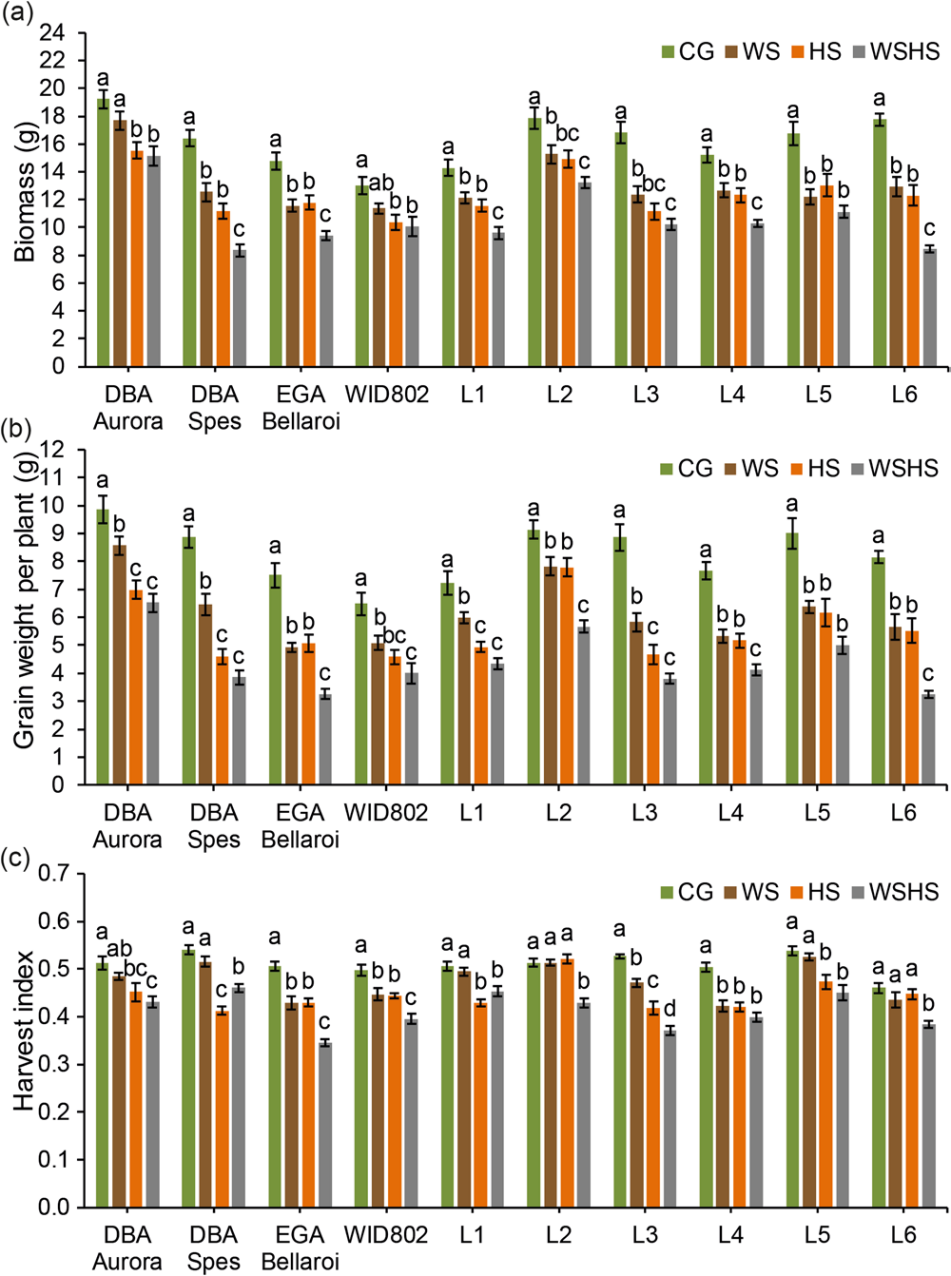
Biomass **(a)**, grain weight per plant **(b)** and harvest index **(c)** of ten durum wheat genotypes in four treatment groups. Means ± SE for *n* = 6 are shown. Different letters (a–c) denote statistically significant differences at the *P* < 0.05 level among treatment groups. CG, control group; WS, water stress group; HS, heat stress group; WSHS, combined water stress and heat stress group.

Biomass (Fig. 4a) was significantly reduced under WS, HS and WSHS compared with CG for all ten genotypes (except for DBA Aurora and WID802 under WS). Biomass under WSHS was significantly lower than both single stress groups in five genotypes (DBA Spes, EGA Bellaroi, L1, L4 and L6). The highest reduction rate of biomass across ten genotypes (relative to each genotype’s control) was observed in L6 under WS (−27.3%), L3 under HS (−33.8%) and L6 under WSHS (−52.5%) (Supplementary Table S3). Grain weight per plant (Fig. 4b) was significantly reduced under all stress treatments for all genotypes. Grain weight under WSHS was significantly lower than both single stress groups in four genotypes (EGA Bellaroi, L2, L4 and L6). For DBA Aurora, DBA Spes, L1 and L3; HS and WSHS were significantly lower than WS. The highest reduction rate of grain weight was observed in EGA Bellaroi under WS (−34.2%), DBA Spes under HS (−48.0%) and L6 under WSHS (−60.4%) (Supplementary Table S3). Harvest index (Fig. 4c) was significantly reduced under all stress treatments for four genotypes (EGA Bellaroi, WID802, L3 and L4). The highest reduction rate of harvest index was observed in L4 under WS (−16.4%), DBA Spes under HS (−23.7%) and EGA Bellaroi under WSHS (−31.7%) (Supplementary Table S3).

**Figure 5:**
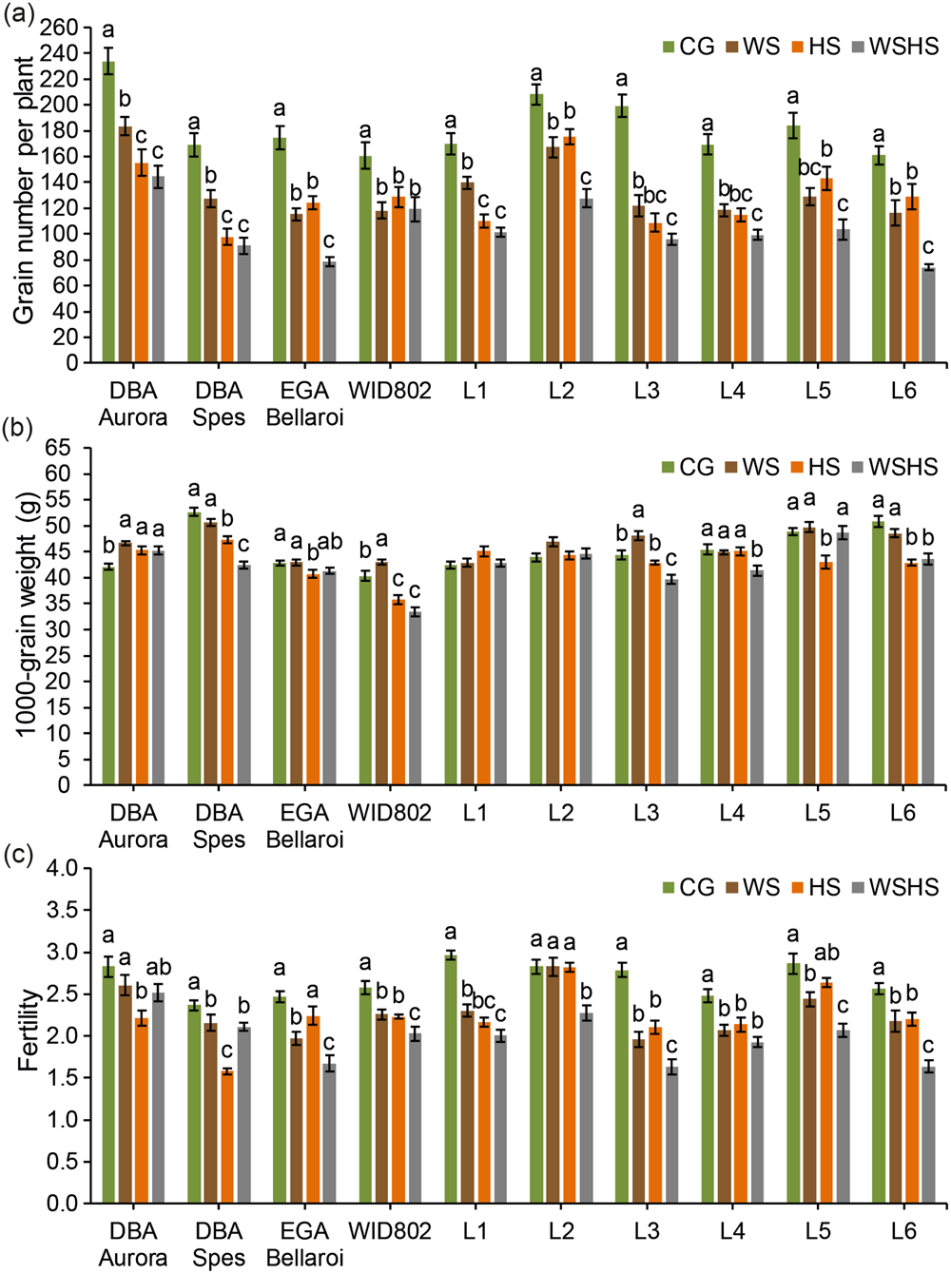
Grain number per plant **(a)**, 1000-grain weight **(b)** and fertility **(c)** of ten durum wheat genotypes in four treatment groups. Means ± SE for *n* = 6 are shown. Different letters (a–c) denote statistically significant differences at the *P* < 0.05 level among treatment groups. When no letters were shown there was no statistical difference (*P* > 0.05) detected among the treatment groups. CG, control group; WS, water stress group; HS, heat stress group; WSHS, combined water stress and heat stress group.

Grain number per plant (Fig. 5a) was significantly reduced under all stress treatments for all genotypes. For three genotypes (EGA Bellaroi, L2 and L6), WSHS had significantly lower grain number than both WS and HS. WID802 showed no significant difference in grain number between the stressed groups. The highest reduction rate of grain number was observed in L3 under WS (−39.0%), L3 under HS (−45.5%) and EGA Bellaroi under WSHS (−55.0%) (Supplementary Table S3). For 1000-grain weight (Fig. 5b), the response is quite different across the genotypes. For DBA Aurora, all stress treatments resulted in significantly higher 1000-grain weight than the control. For WID802 and L3, the highest 1000-grain weight was under WS. For L6, HS and WSHS caused significantly lower 1000-grain weight than CG and WS. All treatments had no significant influence on the 1000-grain weight for L1 and L2. Fertility (Fig. 5c) was significantly reduced under all stress conditions for six genotypes (DBA Spes, WID802, L1, L3, L4 and L6). For DBA Aurora, only HS caused a significant reduction while for L2 it was only WSHS. The highest reduction rate of fertility was observed in L3 under WS (−29.8%), DBA Spes under HS (−33.3%) and L3 under WSHS (−41.3%) (Supplementary Table S3).

**Figure 6:**
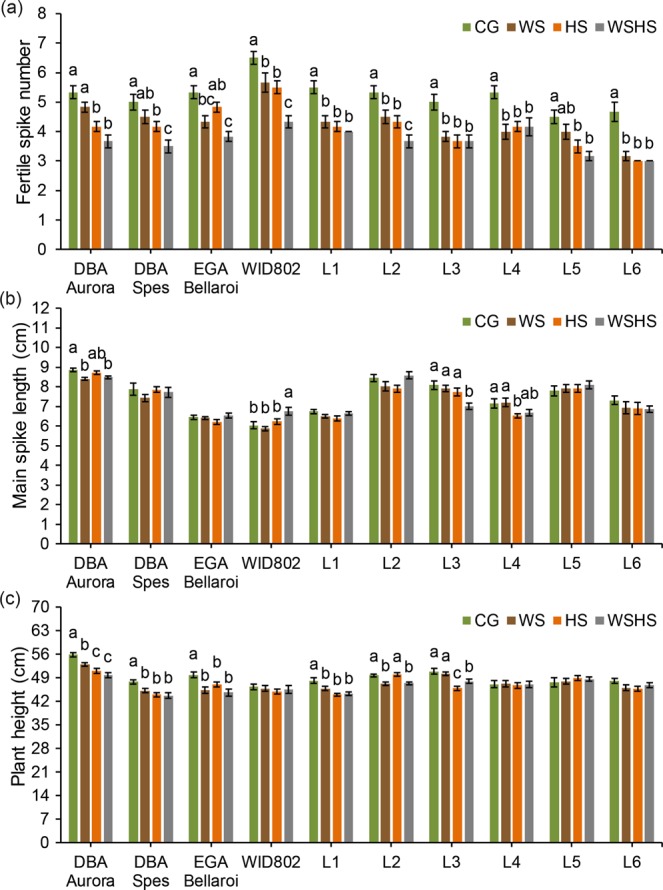
Fertile spike number **(a)**, main spike length **(b)** and plant height **(c)** of ten durum wheat genotypes in four treatment groups. Means ± SE for *n* = 6 are shown. Different letters (a–c) denote statistically significant differences at the *P* < 0.05 level among treatment groups. When no letters were shown there was no statistical difference (*P* > 0.05) detected among the treatment groups. CG, control group; WS, water stress group; HS, heat stress group; WSHS, combined water stress and heat stress group.

Fertile spike number per plant (Fig. 6a) was significantly reduced under all stress treatments for six genotypes (WID802, L1, L2, L3, L4 and L6). No difference was found between CG and WS for DBA Aurora, DBA Spes and L5. For EGA Bellaroi, there was no significant difference between CG and HS. L6 had the highest reduction rate of fertile spike number under all stress conditions (−32.1% under WS, −35.7% under HS and −35.7% under WSHS) (Supplementary Table S3). For the two morphological traits measured, genotype-dependent responses were observed. Six genotypes showed no statistical difference in main spike length among all groups (Fig. 6b). WID802 had the greatest main spike length under WSHS while L3 had the least under WSHS. For plant height (Fig. 6c), stress treatments had no impact in four genotypes (WID802, L4, L5 and L6). DBA Spes, EGA Bellaroi and L1 showed the same pattern where CG had the highest plant height. For L2, WS and WSHS had shorter plant height than CG and HS. Five other yield-related traits, including weight of all spikes per plant, spike harvest index, spikelet number per plant, grain number per spike and spikelet number per spike were also analysed (data available in Supplementary Fig. S4). Notably, the weight of all spikes per plant was significantly reduced (*P* < 0.05) under all stress treatments for all genotypes, suggesting the sensitivity of the trait to water-deficiency and heat stress.

In the ranking summation table based on these five major yield traits, genotypes with a higher index would have had a lower yield reduction rate and are likely to be stress-tolerant. Genotypes with a lower rank summation index had a higher reduction in yield traits and are likely to be stress-sensitive (Table 1). However, index summation provides the ranking of yield reduction rates, without an association of the significant differences between the genotypes. For comparison of the genotypes, *P* values and l.s.d. values for each yield trait are provided in Supplementary Table S3. The highest ranking genotype, DBA Aurora, is stress-tolerant with significantly higher biomass, grain weight, grain number and fertility than all the genotypes under WSHS (Supplementary Table S3). The lowest ranking genotype L6 is stress-sensitive, with the lowest fertility (although not significantly different to EGA Bellaroi and L3) and the lowest fertile tiller number (not significantly different to DBA Spes and L5) under WSHS among all genotypes (Supplementary Table S3).

### Impacts of different stress treatments on grain quality traits are genotype-dependent

**Figure 7:**
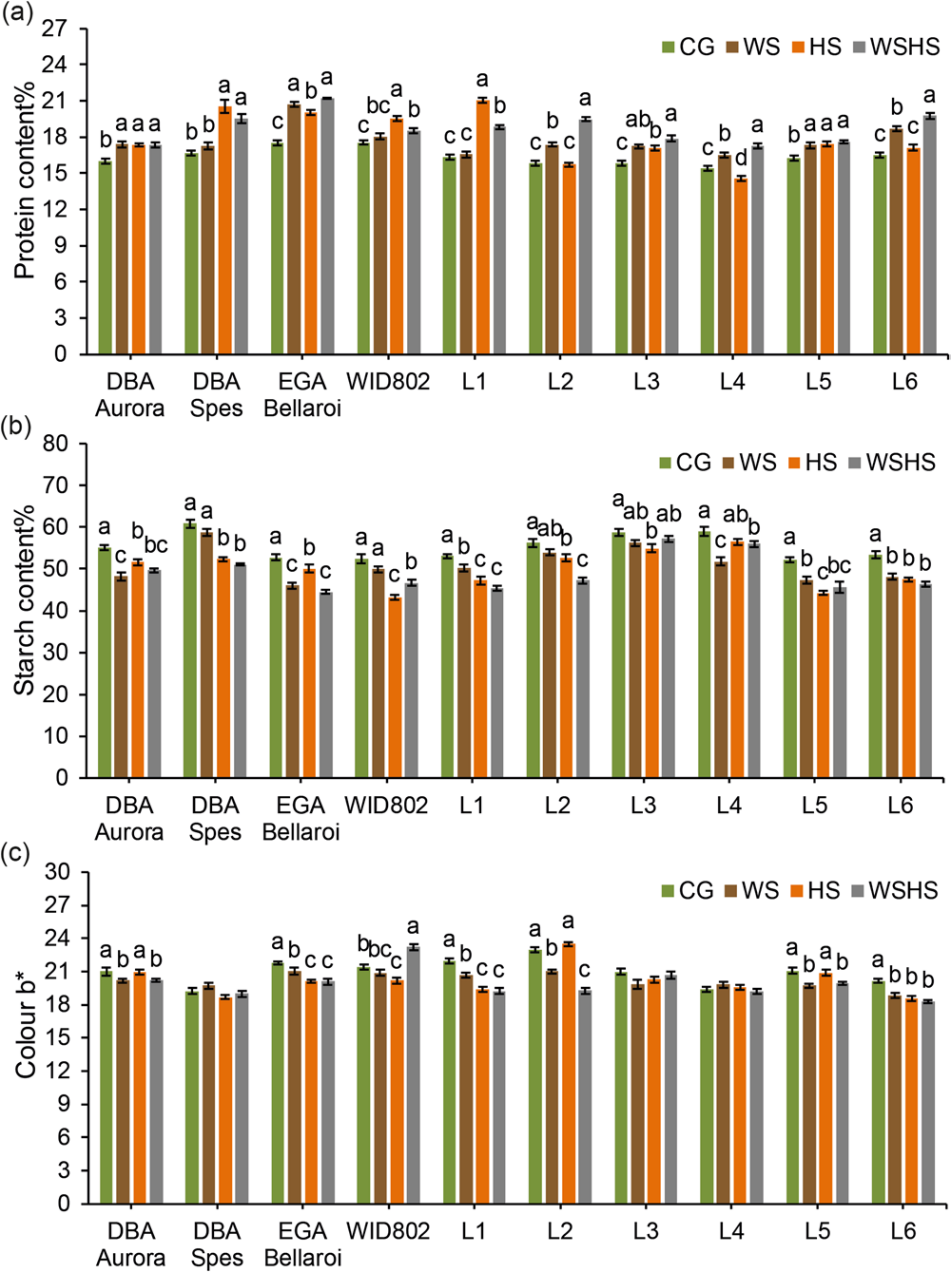
Grain quality attributes of ten durum wheat genotypes in four treatment groups: **(a)** protein content, **(b)** total starch content and **(c)** flour yellowness (+b*). Means ± SE for *n* = 4 are shown. Different letters (a–c) denote statistically significant differences at the *P* < 0.05 level among treatment groups. When no letters were shown there was no statistical difference (*P* > 0.05) detected among the treatment groups. CG, control group; WS, water stress group; HS, heat stress group; WSHS, combined water stress and heat stress group.

For protein content (Fig. 7a), the impact of stress was variable across the genotypes. For four genotypes (DBA Aurora, EGA Bellaroi, L3 and L5), all stress treatments induced significantly greater protein content than CG. For DBA Spes, HS and WSHS had significantly more protein content than CG and WS. For WID802 and L1, the highest protein content was induced by HS, while for L2, L4 and L6 it was WSHS. Five genotypes showed a decrease in starch content (Fig. 7b) under the three stress treatments (DBA Aurora, EGA Bellaroi, L1, L5 and L6). For DBA Spes, WID802 and L2, no difference was found between CG and WS. For L4, no difference was found between CG and HS. Stress treatments caused a significant decrease in flour colour b* (Fig. 7c) in three genotypes (EGA Bellaroi, L1 and L6). For DBA Spes, L3 and L4, stress did not affect the flour yellowness. For DBA Aurora, L2 and L5, the CG and HS treatments resulted in similar b* values. WID802 showed the highest b* value under WSHS.

**Figure 8:**
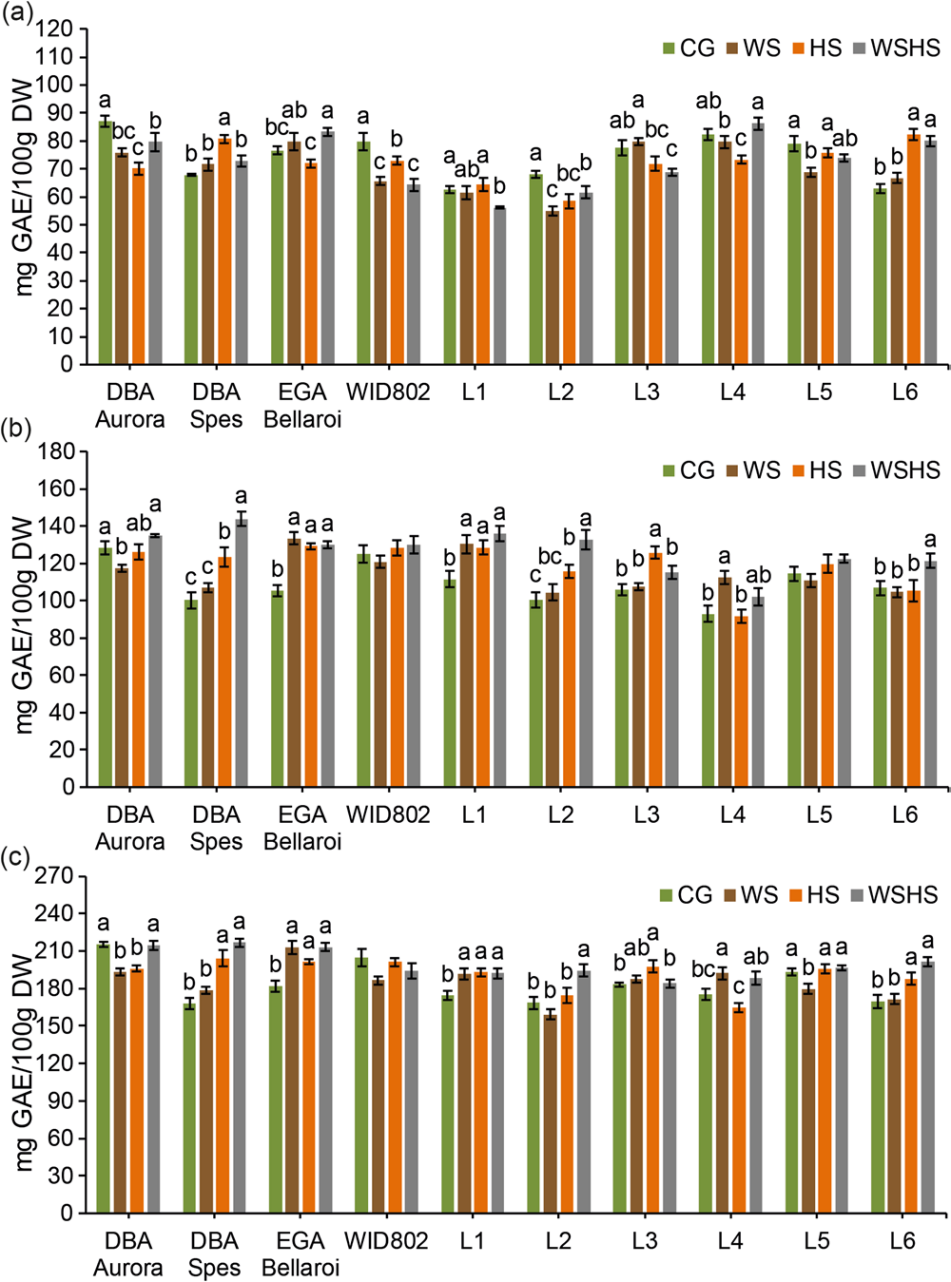
Free **(a)**, bound **(b)** and total **(c)** phenolic content of wholemeal flour of ten durum wheat genotypes in four treatment groups. Means ± SE for *n* = 4 are shown. Different letters (a–c) denote statistically significant differences at the *P* < 0.05 level among treatment groups. When no letters were shown there was no statistical difference (*P* > 0.05) detected among the treatment groups. CG, control group; WS, water stress group; HS, heat stress group; WSHS, combined water stress and heat stress group.

For free, bound and total phenolic content in the grains, the response under different stress treatments was dependent on the genotype. Three genotypes had significantly lower free phenolic content (Fig. 8a) under all stress treatments (DBA Aurora, WID802 and L2). For DBA Spes, HS induced the highest free phenolic content while for L6 it was both HS and WSHS. For bound phenolic content (Fig. 8b), EGA Bellaroi and L1 had the same pattern where all stress treatments induced higher bound phenolic content than in the CG. For L6, the highest bound phenolic content was in WSHS while for L3 it was found in HS. For total phenolic content (Fig. 8c), DBA Spes and L6 showed the same pattern, where higher total phenolic content was found when heat stress was present. For EGA Bellaroi and L1, all three stress treatments increased the total phenolic content with no difference among them. The total phenolic content of WID802 was not impacted by any stress.

### Complex regulatory patterns of stress-responsive microRNAs and their targets

**Figure 9:**
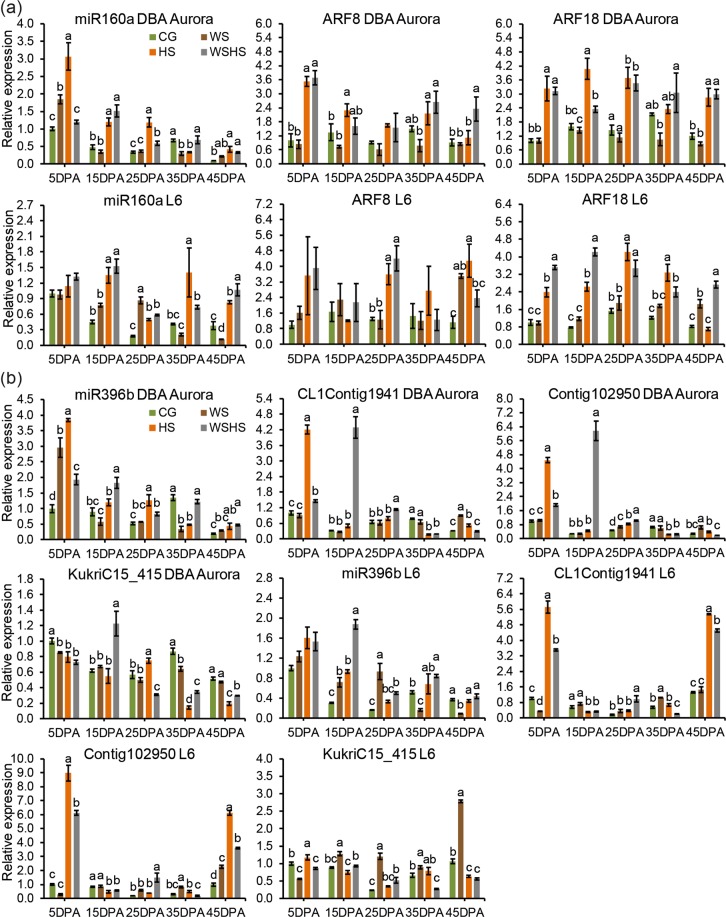
The expression patterns of microRNAs and their targets in the flag leaf of two durum wheat genotypes at five time-points in four treatment groups: **(a)** miR160a and two *ARF*s, **(b)** miR396b and three *HSP*s. Different letters (a–d) denote statistically significant differences at the *P* < 0.05 level among treatment groups within a time-point. When no letters were shown there was no statistical difference (*P* > 0.05) detected among the treatment groups. DPA, days post anthesis. CG, control group; WS, water stress group; HS, heat stress group; WSHS, combined water stress and heat stress group.

Durum miR160 targets two *ARF*s (*ARF8* and *ARF18*) and miR396 targets three *HSP90* genes (CL1Contig1941, Contig102950, and KukriC15_415). The miRNAs and their targets have exhibited complex regulatory patterns in the stress-tolerant genotype DBA Aurora and the stress-sensitive genotype L6 (Fig. 9). For DBA Aurora, WS induced the expression of miR160 at 5DPA, but reduced its expression at 35DPA (Fig. 9a). Under HS, miR160 was upregulated at all time-points except for 35DPA. WSHS did not induce any change of miR160 at 5DPA and 35DPA, but an upregulation was observed at all other time-points. *ARF8* and *ARF18* exhibited very similar expression patterns in DBA Aurora (Fig. 9a). Both HS and WSHS induced significantly higher expression of the *ARFs* than CG and WS at 5DPA, for *ARF18* it also had the same pattern at 25DPA and 45DPA. For L6, no significant difference was observed among treatments at 5DPA for the expression of miR160 and *ARF8*. Similar to DBA Aurora, both HS and WSHS resulted in significantly higher expression than CG and WS at 15DPA for miR160, and 25DPA for the *ARF*s.

For miR396 expression in DBA Aurora, HS induced the highest level at 5DPA and 25DPA (Fig. 9b). WSHS induced the higher miR396 expression at 15DPA. For the *HSP90*s, CL1Contig1941 and Contig102950 exhibited similar expression patterns in DBA Aurora (Fig. 9b). HS induced very high expression of both genes at 5DPA, and WSHS at 15DPA. For KukriC15_415, all stress treatments led to a significant reduction at 5DPA and 35DPA in DBA Aurora. However, significant upregulation of KukriC15_415 was observed under WSHS at 15DPA and HS at 25DPA. For miR396 expression in L6, no difference was found at 5DPA (Fig. 9b). The highest expression of miR396 was found under WSHS at 15DPA and under WS at 25DPA. At 45DPA, miR396 was downregulated only by WS. In L6, CL1Contig1941 and Contig102950 showed significantly higher expression under HS and WSHS at 5DPA and 45DPA, to a greater extent than DBA Aurora. For KukriC15_415, the expression pattern was quite different at each time-point, similar to its expression in DBA Aurora. A significant increase of KukriC15_415 expression was noted at 25DPA and 45DPA under the WS treatment.

### Significant correlations between leaf physiological traits and yield components

To understand the physiological basis of yield performance under stress and possibly assist future selection strategies in breeding programs, the correlation coefficients between leaf physiological traits and major yield components were determined (Table 2). Overall, significant medium to strong positive correlations were found between the three physiological traits measured at different time-points and the four major yield components. The correlation coefficients ranged between 0.514 and 0.833 (*P* < 0.01) but at which time-point the correlation was strongest, differed. For example, the strongest correlation between biomass and chlorophyll content was found for the measurements made at 45DPA (*r* = 0.6398). For stomatal conductance, the strongest correlation with biomass was found for the measurements made at 25DPA (*r* = 0.7007). The highest correlation coefficient reported overall was between grain weight per plant and leaf relative water content at 5DPA (*r* = 0.8333).

**Table 2:** Correlation coefficients (*r*) between yield components and physiological traits at different time-points in ten durum wheat genotypes. CC, chlorophyll content; SC, stomatal conductance; RWC, leaf relative water content; DPA, days post anthesis.

	CC 5DPA	CC 15DPA	CC 25DPA	CC 35DPA	CC 45DPA	SC 5DPA	SC 15DPA	SC 25DPA	SC 35DPA	SC 45DPA	RWC 5DPA	RWC 15DPA	RWC 25DPA	RWC 35DPA	RWC 45DPA
Biomass	0.6084	0.6143	0.6252	0.6196	**0.6398**	0.6993	0.6984	**0.7007**	0.6856	0.6882	0.7877	**0.8003**	0.7969	0.6687	0.6406
Grain Weight	0.6371	0.6568	0.6664	0.6880	**0.7121**	**0.7567**	0.7516	0.7537	0.7505	0.7474	**0.8333**	0.8206	0.8163	0.7559	0.7239
Harvest Index	0.5143	0.5531	0.5570	0.6305	**0.6607**	0.6165	0.6090	0.6022	**0.6215**	0.6102	**0.7498**	0.6663	0.6546	0.7436	0.7055
Grain Number	0.6226	0.6307	0.6311	**0.6323**	0.6128	**0.7424**	0.7380	0.7422	0.7339	0.7228	0.8166	**0.8196**	0.8106	0.7661	0.6855

Correlation of CC and SC with yield components is based on individual values of six biological replicates from each of the four treatment groups, at each of the five time-points per genotype (*n* = 240). Correlation of RWC with yield components is based on the mean values of the replicates from each treatment group at each time-point per genotype (*n* = 40). All correlations were statistically significant (*P* < 0.01). The highest correlation coefficient between a yield component and each physiological trait is highlighted in bold.

## Discussion

Flowering and grain filling are critical periods of cereal reproduction. Both water deficiency and high temperatures during these critical periods are known to cause significant yield loss in cereals^4,13,46,47^. Previous studies have shown that the combination of two stresses is often detrimental, causing significantly more yield reduction than a single stress alone^4,16,48,49^. In the present study, the effects of stress combination were more pronounced on certain yield traits. However, the effects were not necessarily additive and it depended upon the genotype. For example, even though all stress treatments have led to a significant reduction in grain number for all genotypes, the effect of a single HS treatment was more severe than a single WS treatment in several genotypes (Fig. 5b), with no greater impact observed with the combined stress treatment. DBA Aurora and DBA Spes also showed more sensitivity to HS in terms of spike fertility. Elevated night-time temperature (≥20 °C) has been reported to significantly decrease fertility and grain number in bread wheat^50^. The reduction was caused by embryo abortion due to limited photosynthetic activity under heat stress^50^. However, for genotypes like L2 and L6, where WS and HS only caused a moderate reduction in grain number and fertility, the effects of these stresses combined were more severe. This was most likely due to the possible synergistic interactions of two types of stress and its implication on photosynthetic activity that affect seed-set and embryo development^4,51^. These results justify the need to evaluate specific yield traits under different targeted environmental conditions in the field. Moreover, although controlled environments can provide valuable experimental data where a specific stress condition can be targeted, the limitations in extrapolating pot-based experiments to crop performance in the field must be acknowledged^52^. This is especially the case for water stress, due to the relatively low water retention capacity in pot trials that may not conclusively represent field conditions where stress can slowly develop over a sustained period of time. For experiments conducted in controlled environments, soil water content in pots must be monitored frequently to prevent intense stress shock that is unintended. Moreover, for breeding and selection purposes, larger-scale field trials need to be incorporated to evaluate yield performance and to select for stress-tolerant genotypes suited for the Australian wheat belt.

The grain traits measured in this study varied over a wide range in individual genotypes under different stress conditions. High protein content is crucial to ensure the development of high quality end-use products in durum^1,53^. Reports in both durum and bread wheat have shown that high temperature during grain filling can significantly increase grain protein content^25,48,54^. However, such increases in protein content were not necessarily associated with better grain quality. In this study, the presence of heat stress (HS and WSHS) has led to higher protein content in several genotypes (such as DBA Spes and L1). However, heat stress also induced a higher reduction of total starch content in these genotypes. The increase of grain protein content under heat stress could be attributed to the reduced 1000-grain weight observed, which is consistent with previous reports where higher protein content was associated with lower grain size under stress^22,49^, thus the overall accumulation of protein in the grains was not actually improved. However, we have identified durum genotypes in which the grain quality traits were not influenced by stress. For example, protein content of L2 and L6 remained similar to the control under HS. The reduction of starch content in L2 under HS was also minimal (6.4%). Moreover, none of the stress treatments had any significant impact on the 1000-grain weight of L2. Furthermore, stress could even induce certain grain traits that are possibly beneficial in some genotypes. Bioactive compounds like phenolic compounds in wholegrain products present health-promoting benefits when consumed^45,55^. Previous reports have also suggested that the antioxidant properties of phenolic acids could be associated with the stress tolerance capacity through contributions towards ROS scavenging^56,57^. Here, significant increases of free, bound and total phenolic content under stress were noted, specific to certain genotypes. The highest increase was recorded for DBA Spes, where its total phenolic content increased by 29% under WSHS. The accumulation of yellow-amber pigmentation in the grains is another favourable quality trait for durum. High yellow pigmentation adds the nutritional value (mainly from carotenoids) and aesthetic appeal in marketing end-use products^58,59^. Here, how flour yellowness was influenced by stress depended upon the stress type and genotype. For example, under WSHS, colour b* of WID802 significantly increased by 8.4% but for L1 and L2, a significant decline was observed when exposed to combined stress (−12.5% and −16.0%, respectively). Such phenomena is consistent with previous reports where flour pigmentation showed dependence on the genotype being investigated^48,49^. The results provide new knowledge on the selected Australian germplasm when considering breeding for combined high grain quality traits and improved yield under stressed environments.

In the leaf tissue, photosynthetic activity, transpiration and cellular water status are all very sensitive to water deficiency and high temperature. These leaf traits are the first affected during stress^13,24,60,61^. In this study, substantial variation was observed for leaf physiological traits under single and combined stress across the durum genotypes. The influence of stress depends on the time-point of measurement (thus length of stress) and genotype. Previous reports have suggested that combined water-deficit and heat stress usually have a much more severe impact on the physiological traits than a single stress alone^4,62^. In our study, whether combined stress was more influential was genotype-dependent. Combined stress had greater impact on the chlorophyll content than a single stress in the genotypes that were more sensitive to stress, such as L6 and L3. For genotypes that are more stress-tolerant like DBA Aurora, chlorophyll content under combined stress showed no difference to at least one single stress treatment. Furthermore, even though stomatal conductance decreased significantly under all stress, it was inhibited to a greater extent in stress-sensitive genotypes (L6 and L3). Similarly, DBA Aurora maintained a higher percentage of RWC (even at 45DPA) than stress-sensitive genotypes. The performance of stress-tolerant genotypes could be attributed to the coordination of physiological responses. The importance of maintaining cellular turgidity (as reflected by RWC) required for plant growth and survival is widely recognized^13,24,63,64^. Stomatal conductance, which determines the CO_2_ and water vapour diffusion in/out of the leaf, indicates the regulation and control of photosynthetic and transpiration responses in response to stress^24,65^. The reduction of stomatal conductance in stress-tolerant genotypes could help to maintain cellular water turgidity, but not as much as in the stress-sensitive genotypes where transpiration activity and carbon dioxide supply would be severely inhibited. Thus the photosynthetic capacity could be maintained in stress-tolerant genotypes. Maintained leaf RWC, and subsequently less damage, would likely allow the chlorophyll content to be maintained leading to greater photosynthetic capacity^66,67^. Efficient photosynthesis under abiotic stress has a decisive role in the development of reproductive tissues, the accumulation of reserve nutrients and may also directly affect the final grain yield^4^. The reduced inhibition of stomatal conductance and the maintenance of RWC and chlorophyll content that we observed in stress-tolerant varieties, likely reduced the negative impacts on the reproductive organs contributing to the maintenance of grain number and grain weight at harvest. Thus, leaf traits associated with better physiological performance are often used in phenotyping techniques for making breeding decisions^68,69^. Here, we found moderate to strong positive correlations between leaf physiological traits and yield components. Interestingly, the strongest correlation coefficients were found at different time-points for each physiological trait/yield component combination. Chlorophyll content had a stronger correlation with yield components at later developmental stages (35DPA or 45DPA), whereas the correlations between RWC and the yield components were the strongest at early stages of grain development (5DPA or 15DPA). For stomatal conductance, time-points with the strongest correlation varied but the strongest correlation was at 5DPA with grain weight, suggesting that the coordinating role of stomatal conductance could be of great importance at the start of grain formation. Our results indicated that germplasm screening under water-deficit and heat stress conditions needs to consider the timing of physiological phenotyping to better predict yield performance, based on the type of trait used. However, due to the limitations of pot-based experiments, investigation under field conditions on a larger scale needs to be taken to further inform strategic trait-based crossing and selection in breeding.

In this study, the response patterns of durum miRNAs and their targets were dependant on the genotype, the time-point of grain development and the stress type. We have experimentally validated that under water stress that occurred before flowering, durum miR160 specifically targets *ARF8* and *ARF18*, both crucial regulators of many auxin-mediated processes within plant development and stress adaptation^24,38^. However, in this study, no significant changes of *ARFs* could be found under water-deficit stress (except for 45DPA in L6). This suggests that miR160/*ARFs*-mediated water stress responses are predominantly activated at early reproductive stages rather than later during grain filling. Interestingly, the miR160-*ARFs* signalling pathways were activated when heat stress was present. Significant increases in *ARF* expression were found under HS and WSHS. miR160 was also upregulated by heat at several time-points investigated. These results are consistent with studies in bread wheat and barley, where miR160 was heat-induced^70,71^. Transcriptome analysis in bread wheat has also revealed that high temperature stress can activate auxin signal pathways involving multiple *ARFs* and AUX/IAA proteins^72^. Such patterns could contribute to heat stress tolerance through auxin signalling and positive response of auxin homeostasis, contributing to the adaptive traits observed at the physiological level such as maintained photosynthetic activity^72^.

Here, we also reported the first expression profiling of durum miR396-*HSPs* under stress during reproduction. Similar to previous studies^73–76^, the expression patterns depended upon the genotype and timing. A significant increase of miR396 was found in DBA Aurora at a very early stage (5DPA), where such responses occurred in L6 at 15DPA. Two *HSP90*s were significantly induced at early reproductive stages in both genotypes when heat stress was present. HSPs assist other proteins to fold properly, prevent unwanted denaturation, and maintain their functional state under elevated temperatures^41,77^. Thus stress-induced HSPs in durum could assist in protecting cellular protein stability against heat. In addition, HSPs play a role in protecting the photosynthetic electron transport chain to prevent damage in the chloroplasts^78,79^, which would help maintain high chlorophyll content under stress. The links between durum miR396-*HSPs* and phenotypic performance require further investigation with specific focus on photosynthetic thermotolerance. The expression patterns observed in this study have also confirmed that the regulatory relationships between miRNAs and the targets are not one-on-one specific. Feedback loops will contribute to underpin the homeostasis of miRNA and target levels, together with other regulating factors^32,37^. To fully elucidate the epigenetic basis in durum, a high-throughput study of the whole miRNA transcriptome using Australian germplasm is currently underway. Such complex studies have been undertaken, knowing that the genome assembly of durum wheat^80^ and the fast development of next-generation technologies are now readily available.

In conclusion, we have identified Australian durum genotypes with high resilience and productivity specific to each stress, and the stress-adaptive traits associated with their production capacity. The impacts of high temperature and water deficiency on the productivity and quality highly depend on the genotype and the combination of stress. While the traits studied provide valuable information towards understanding reproductive-stage stress response under controlled environments, future validation under field conditions will no doubt bring additional opportunities for germplasm evaluation and selection in breeding. Moreover, while epigenetic breeding is in its infancy, the precise integration of desired molecular traits along with favourable agronomic traits will certainly provide new strategies to develop superior germplasm.

## Supplementary information

Supplementary information

## Data Availability

All data generated or analysed during this study are included in this article (and its Supplementary Information files).
