# Supplementary material for: Genotypic performance of Australian durum under single and combined water-deficit and heat stress during reproduction
Source: Sci Rep. 2019 Oct 18;9:14986. doi: 10.1038/s41598-019-49871-x (PMC6802220; doi:10.1038/s41598-019-49871-x)

Genotypic performance of Australian durum under single and combined water-deficit and heat stress during reproduction

Haipei Liu[1]*, Amanda J. Able[1], Jason A. Able[1]

[1] School of Agriculture, Food & Wine, Waite Research Institute, The University of Adelaide, Urrbrae, SA, Australia

Supplementary Materials

Supplementary Table S1. Durum wheat varieties and University of Adelaide breeding lines used in this study and their abbreviation.

Genotype	Abbreviation	Information
DBA Aurora	N/A	Commercial variety, release from South Australia
DBA Spes	N/A	Commercial variety, release from South Australia
EGA Bellaroi	N/A	Commercial variety, release from New South Wales
WID802	N/A	Commercial variety, release from South Australia
UAD1151101	L1	Advanced DBA (Durum Breeding Australia) breeding line at University of Adelaide
UAD1154197	L2	Advanced DBA breeding line at University of Adelaide
UAD1301011-4	L3	Advanced DBA breeding line at University of Adelaide
UAD1301012-12	L4	Advanced DBA breeding line at University of Adelaide
UAD1301018-6	L5	Advanced DBA breeding line at University of Adelaide
UAD1301020-8	L6	Advanced DBA breeding line at University of Adelaide

Supplementary Table S2. Rank summation index of ten durum genotypes based on their sensitivity to single water-deficit stress and single heat stress.

**Water-deficit stress**						
Genotype	Biomass	Grain weight	Harvest index	Grain number per plant	1000-grain weight	**Summation Index**
DBA Aurora	10	10	6	8	10	**44**
L2	8	9	10	9	7	**43**
L1	7	8	9	10	5	**39**
WID802	9	7	4	6	8	**34**
DBA Spes	4	6	7	7	2	**26**
L5	2	5	8	3	6	**24**
L3	3	2	3	1	9	**18**
L4	6	4	1	4	3	**18**
L6	1	3	5	5	1	**15**
EGA Bellaroi	5	1	2	2	4	**14**
**Heat stress**						
Genotype	Biomass	Grain weight	Harvest index	Grain number per plant	1000-grain weight	**Summation Index**
L2	10	10	10	10	8	**48**
DBA Aurora	7	9	7	4	10	**37**
WID802	5	8	8	8	3	**32**
L1	8	6	4	3	9	**30**
L4	9	3	3	5	7	**27**
L6	3	5	9	9	1	**27**
EGA Bellaroi	6	4	5	6	5	**26**
L5	4	7	6	7	2	**26**
L3	1	2	2	1	6	**12**
DBA Spes	2	1	1	2	4	**10**

Supplementary Table S3. Yield traits and reduction rate of ten durum wheat genotypes under water stress (WS); heat stress (HS) and combined water and heat stress (WSHS). Green highlights the lowest reduction rate across ten genotypes, while red highlights the highest reduction rate across ten genotypes. *P* value and l.s.d. is shown across genotypes for each trait under a given treatment. ***** indicates statistical significance for the changes of yield traits under stress when compared with the control ($P < 0.05$).

**Biomass**	DBA Aurora	DBA Spes	EGA Bellaroi	WID802	L1	L2	L3	L4	L5	L6	P value	l.s.d.
CG	19.217	16.410	14.792	13.022	14.280	17.868	16.818	15.203	16.725	17.762	<0.001	1.874
WS	17.683	12.533	11.572	11.370	12.122	15.270	12.382	12.665	12.173	12.917	<0.001	1.605
HS	15.517	11.185	11.778	10.347	11.553	14.938	11.137	12.330	13.017	12.283	<0.001	1.703
WSHS	15.158	8.355	9.393	10.057	9.595	13.215	10.235	10.287	11.122	8.433	<0.001	1.301
WS reduction rate	-7.98%	-23.62%*	-21.77%*	-12.68%	-15.11%*	-14.54%*	-26.38%*	-16.70%*	-27.21%*	-27.28%*	N/A	N/A
HS reduction rate	-19.25%*	-31.84%*	-20.37%*	-20.54%*	-19.09%*	-16.40%*	-33.78%*	-18.90%*	-22.17%*	-30.84%*	N/A	N/A
WSHS reduction rate	-21.12%*	-49.09%*	-36.50%*	-22.77%*	-32.81%*	-26.04%*	-39.14%*	-32.34%*	-33.50%*	-52.52%*	N/A	N/A

**Grain weight**	DBA Aurora	DBA Spes	EGA Bellaroi	WID802	L1	L2	L3	L4	L5	L6	P value	l.s.d.
CG	9.853	8.873	7.505	6.482	7.235	9.145	8.863	7.663	9.010	8.158	<0.001	1.171
WS	8.568	6.462	4.940	5.080	5.985	7.828	5.835	5.337	6.377	5.647	<0.001	0.873
HS	6.983	4.613	5.072	4.587	4.943	7.785	4.677	5.168	6.177	5.523	<0.001	0.932
WSHS	6.517	3.855	3.252	3.997	4.343	5.670	3.807	4.110	5.010	3.230	<0.001	0.701
WS reduction rate	-13.04%*	-27.18%*	-34.18%*	-21.63%*	-17.28%*	-14.40%*	-34.17%*	-30.36%*	-29.23%*	-30.79%*	N/A	N/A
HS reduction rate	-29.13%*	-48.01%*	-32.42%*	-29.24%*	-31.67%*	-14.87%*	-47.24%*	-32.56%*	-31.45%*	-32.30%*	N/A	N/A
WSHS reduction rate	-33.86%*	-56.56%*	-56.67%*	-38.34%*	-39.97%*	-38.00%*	-57.05%*	-46.37%*	-44.40%*	-60.41%*	N/A	N/A

Supplementary Table S3 continued.

**Harvest index**	DBA Aurora	DBA Spes	EGA Bellaroi	WID802	L1	L2	L3	L4	L5	L6	P value	l.s.d.
CG	0.512	0.540	0.506	0.497	0.505	0.513	0.526	0.504	0.538	0.460	<0.001	0.0283
WS	0.485	0.515	0.429	0.446	0.494	0.513	0.471	0.422	0.525	0.434	<0.001	0.0316
HS	0.451	0.412	0.430	0.443	0.428	0.521	0.418	0.419	0.473	0.447	<0.001	0.0321
WSHS	0.430	0.460	0.345	0.395	0.453	0.429	0.371	0.398	0.449	0.383	<0.001	0.0299
WS reduction rate	-5.34%	-4.58%	-15.24%*	-10.28%*	-2.17%	-0.01%	-10.58%*	-16.41%*	-2.34%	-5.51%	N/A	N/A
HS reduction rate	-11.81%*	-23.71%*	-15.05%*	-10.81%*	-15.19%*	1.66%	-20.57%*	-16.86%*	-12.12%*	-2.71%	N/A	N/A
WSHS reduction rate	-15.96%*	-14.87%*	-31.72%*	-20.52%*	-10.29%*	-16.35%*	-29.42%*	-20.99%*	-16.47%*	-16.75%*	N/A	N/A

**Grain number per plant**	DBA Aurora	DBA Spes	EGA Bellaroi	WID802	L1	L2	L3	L4	L5	L6	P value	l.s.d.
CG	234.00	169.00	174.83	160.83	170.17	208.17	199.33	169.33	184.17	161.00	<0.001	25.12
WS	183.50	127.17	115.33	118.17	139.67	167.33	121.67	118.67	128.83	116.50	<0.001	19.46
HS	155.00	97.83	124.33	128.67	110.00	175.50	108.67	114.67	142.83	128.83	<0.001	21.22
WS reduction rate	144.50	90.83	78.67	119.17	101.33	127.67	96.00	99.33	103.67	74.17	<0.001	17.48
HS reduction rate	-21.58%*	-24.75%*	-34.03%*	-26.53%*	-17.92%*	-19.62%*	-38.96%*	-29.92%*	-30.05%*	-27.64%*	N/A	N/A
WSHS reduction rate	-33.76%*	-42.11%*	-28.88%*	-20.00%*	-35.36%*	-15.69%*	-45.48%*	-32.28%*	-22.44%*	-19.98%*	N/A	N/A
WS reduction rate	-38.25%*	-46.25%*	-55.00%*	-25.91%*	-40.45%*	-38.67%*	-51.84%*	-41.34%*	-43.71%*	-53.93%*	N/A	N/A

Supplementary Table S3 continued.

**1000-grain weight**	DBA Aurora	DBA Spes	EGA Bellaroi	WID802	L1	L2	L3	L4	L5	L6	P value	l.s.d.
CG	42.06	52.68	42.84	40.37	42.45	43.98	44.40	45.39	48.84	50.86	<0.001	2.257
WS	46.69	50.72	42.92	43.02	42.90	46.85	48.17	44.92	49.74	48.52	<0.001	2.049
HS	45.32	47.27	40.69	35.74	45.09	44.33	42.91	45.08	43.09	42.86	<0.001	2.338
WS reduction rate	45.26	42.42	41.30	33.46	42.80	44.63	39.64	41.36	48.62	43.60	<0.001	2.595
HS reduction rate	11.02%*	-3.71%	0.18%	6.56%*	1.07%	6.55%	8.47%*	-1.04%	1.83%	-4.59%	N/A	N/A
WSHS reduction rate	7.74%*	-10.27%*	-5.03%*	-11.45%*	6.24%	0.81%	-3.36%	-0.69%	-11.78%*	-15.72%*	N/A	N/A
WS reduction rate	7.60%*	-19.47%*	-3.61%	-17.10%*	0.84%	1.50%	-10.73%*	-8.88%*	-0.44%	-14.28%*	N/A	N/A

**Fertility**	DBA Aurora	DBA Spes	EGA Bellaroi	WID802	L1	L2	L3	L4	L5	L6	P value	l.s.d.
CG	2.83	2.37	2.46	2.58	2.96	2.83	2.79	2.48	2.86	2.57	<0.001	0.242
WS	2.61	2.16	1.97	2.26	2.30	2.83	1.96	2.07	2.44	2.18	<0.001	0.263
HS	2.21	1.58	2.24	2.23	2.16	2.82	2.10	2.14	2.64	2.21	<0.001	0.202
WS reduction rate	2.52	2.11	1.67	2.03	2.01	2.28	1.64	1.93	2.07	1.64	<0.001	0.234
HS reduction rate	-7.80%	-8.89%*	-19.94%*	-12.35%*	-22.29%*	-0.09%	-29.75%*	-16.52%*	-14.71%*	-15.11%*	N/A	N/A
WSHS reduction rate	-21.78%*	-33.31%*	-8.93%	-13.39%*	-26.97%*	-0.43%	-24.51%*	-13.91%*	-7.72%	-13.95%*	N/A	N/A
WS reduction rate	-11.01%	-10.95%*	-32.04%*	-21.35%*	-32.30%*	-19.56%*	-41.28%*	-22.34%*	-27.76%*	-36.21%*	N/A	N/A

Supplementary Table S3 continued.

**Fertile spike number**	DBA Aurora	DBA Spes	EGA Bellaroi	WID802	L1	L2	L3	L4	L5	L6	P value	l.s.d.
CG	5.33	5.00	5.33	6.50	5.50	5.33	5.00	5.33	4.50	4.67	<0.001	0.680
WS	4.83	4.50	4.33	5.67	4.33	4.50	3.83	4.00	4.00	3.17	<0.001	0.646
HS	4.17	4.17	4.83	5.50	4.17	4.33	3.67	4.17	3.50	3.00	<0.001	0.514
WSHS	3.67	3.50	3.83	4.33	4.00	3.67	3.67	4.17	3.17	3.00	<0.001	0.552
WS	-9.38%	-10.00%	-18.75%*	-12.82%*	-21.21%*	-15.63%*	-23.33%*	-25.00%*	-11.11%	-32.14%*	N/A	N/A
HS	-21.88%*	-16.67%*	-9.38%	-15.38%*	-24.24%*	-18.75%*	-26.67%*	-21.88%*	-22.22%*	-35.71%*	N/A	N/A
WSHS	-31.25%*	-30.00%*	-28.13%*	-33.33%*	-27.27%*	-31.25%*	-26.67%*	-21.88%*	-29.63%*	-35.71%*	N/A	N/A

Supplementary Table S4. qPCR primers used in this study.

Gene name	Description	Forward primer (5' to 3')	Reverse primer (5' to 3')
miR160		CTGGCTCCCTGTATGCCAAA	Universal primer provided in the MystiCq microRNA cDNA Synthesis Mix Kit
miR396		CCTTCCACAGCTTTCTTGAACTT	Universal primer provided in the MystiCq microRNA cDNA Synthesis Mix Kit
CL33956Contig1	Auxin response factor 8	CATTATCATCACACCGACAGCTAC	GGGTAAGGTGGAGATCCGATAAA
CL3649Contig1	Auxin response factor 18	CCTATGCTGTTACTCGGACAA	TGAGCACAAAGCCCTTAGGTA
CL1Contig1941	Heat shock protein 90	CCAGCCTCAAGGACTATGTGA	GCTTCTCAAGGAAGGGAGAG
contig102950	Heat shock protein 90	ACTCCACCAACAGGACCAAG	CAGCACCTCATAGCCCTTCT
KukriC15_415	Heat shock protein 90	AGGAGGGGACAGAACGAGAT	TGACCAATGGCATACTCATCA
GAPDH	GAPDH as reference gene	CTTCCAGGGTGACAACAGGT	GTGCTGTATCCCCACTCGTT

Supplementary Table S5. Leaf physiological traits of ten durum wheat genotypes under water stress (WS); heat stress (HS) and combined water and heat stress (WSHS). CC, chlorophyll content; SC, stomatal conductance; RWC, leaf relative water content. DPA, days post anthesis. Statistical significance is shown for *P* value and l.s.d. value (at 5% level) for comparing across time-points within each treatment group for a genotype; and for comparing across four treatment groups within each time-point for a genotype.

**DBA Aurora (CC)**	5DPA	15DPA	25DPA	35DPA	45DPA	F pr. (DPA)	l.s.d.
CG	62.13	60.35	59.08	55.32	50.97	<0.001	1.010
WS	60.42	58.97	57.20	53.90	47.33	<0.001	1.188
HS	61.58	59.10	57.45	51.58	46.28	<0.001	1.074
WSHS	59.60	58.25	56.48	50.97	45.35	<0.001	1.065
F pr. (Treatment)	<0.001	0.009	<0.001	<0.001	<0.001		
l.s.d.	1.177	1.146	0.888	1.059	1.202		

**DBA Spes (CC)**	5DPA	15DPA	25DPA	35DPA	45DPA	F pr. (DPA)	l.s.d.
CG	62.17	61.05	60.02	57.27	55.18	<0.001	0.917
WS	60.37	59.42	57.90	55.48	50.05	<0.001	0.963
HS	61.10	59.63	58.30	55.10	49.52	<0.001	0.927
WSHS	59.35	58.05	56.37	51.35	46.47	<0.001	0.826
F pr. (Treatment)	<0.001	<0.001	<0.001	<0.001	<0.001		
l.s.d.	0.963	0.911	0.794	0.908	1.017		

**EGA Bellaroi (CC)**	5DPA	15DPA	25DPA	35DPA	45DPA	F pr. (DPA)	l.s.d.
CG	61.88	60.82	59.10	56.53	47.33	<0.001	1.412
WS	59.03	57.67	56.35	53.90	42.48	<0.001	0.860
HS	58.82	57.95	56.52	52.45	42.77	<0.001	1.371
WSHS	58.07	56.35	54.08	49.33	39.92	<0.001	1.165
F pr. (Treatment)	<0.001	<0.001	<0.001	<0.001	<0.001		
l.s.d.	1.115	1.043	0.996	1.122	1.753		

**WID802 (CC)**	5DPA	15DPA	25DPA	35DPA	45DPA	F pr. (DPA)	l.s.d.
CG	60.78	59.38	57.22	54.65	46.23	<0.001	1.304
WS	58.77	57.77	55.13	49.88	44.73	<0.001	1.095
HS	60.53	58.03	56.05	52.70	43.78	<0.001	1.061
WSHS	58.52	57.33	54.57	49.45	40.43	<0.001	1.112
F pr. (Treatment)	<0.001	<0.001	<0.001	<0.001	<0.001		
l.s.d.	1.074	0.929	1.145	1.201	1.406		

**L1 (CC)**	5DPA	15DPA	25DPA	35DPA	45DPA	F pr. (DPA)	l.s.d.
CG	59.55	57.68	56.27	54.28	50.43	<0.001	0.939
WS	58.10	55.73	52.97	49.90	42.73	<0.001	1.128
HS	58.97	56.08	53.50	50.75	45.92	<0.001	0.974
WSHS	57.33	55.55	51.82	47.55	40.25	<0.001	1.019
F pr. (Treatment)	<0.001	<0.001	<0.001	<0.001	<0.001		

l.s.d.	0.928	0.963	0.916	1.023	1.278		

**L2 (CC)**	5DPA	15DPA	25DPA	35DPA	45DPA	F pr. (DPA)	l.s.d.
CG	61.80	61.02	59.25	55.75	52.25	<0.001	0.879
WS	60.57	59.43	57.28	53.87	49.77	<0.001	0.888
HS	60.57	59.38	57.63	52.33	48.48	<0.001	0.900
WSHS	59.67	59.02	56.80	51.27	47.38	<0.001	0.842
F pr. (Treatment)	<0.001	<0.001	<0.001	<0.001	<0.001		
l.s.d.	0.878	0.727	0.81	0.953	1.042		

**L3 (CC)**	5DPA	15DPA	25DPA	35DPA	45DPA	F pr. (DPA)	l.s.d.
CG	60.35	58.87	56.92	54.13	49.30	<0.001	0.895
WS	57.65	55.30	53.15	51.72	43.57	<0.001	0.904
HS	58.58	56.28	53.17	50.43	43.80	<0.001	0.807
WSHS	57.17	54.35	52.12	47.20	39.60	<0.001	0.949
F pr. (Treatment)	<0.001	<0.001	<0.001	<0.001	<0.001		
l.s.d.	0.819	0.903	0.891	0.83	1.047		

**L4 (CC)**	5DPA	15DPA	25DPA	35DPA	45DPA	F pr. (DPA)	l.s.d.
CG	60.82	58.20	56.08	53.63	51.23	<0.001	0.886
WS	58.67	55.88	54.07	50.98	47.48	<0.001	0.835
HS	58.32	55.37	53.37	50.10	47.03	<0.001	0.922
WSHS	57.80	54.63	52.47	49.42	45.45	<0.001	1.013
F pr. (Treatment)	<0.001	<0.001	<0.001	<0.001	<0.001		
l.s.d.	0.849	0.931	0.847	0.982	1.019		

**L5 (CC)**	5DPA	15DPA	25DPA	35DPA	45DPA	F pr. (DPA)	l.s.d.
CG	61.35	61.20	60.18	57.60	55.72	<0.001	0.860
WS	58.80	57.02	55.45	52.03	49.35	<0.001	0.866
HS	58.75	57.75	55.98	52.43	48.30	<0.001	0.870
WSHS	57.97	55.95	52.87	50.82	48.12	<0.001	0.934
F pr. (Treatment)	<0.001	<0.001	<0.001	<0.001	<0.001		
l.s.d.	0.862	0.825	0.783	0.966	1.014		

**L6 (CC)**	5DPA	15DPA	25DPA	35DPA	45DPA	F pr. (DPA)	l.s.d.
CG	62.15	60.82	59.17	55.45	50.48	<0.001	0.973
WS	57.95	56.65	54.80	50.68	42.67	<0.001	0.895
HS	60.42	56.85	55.02	50.43	41.85	<0.001	0.890
WSHS	57.88	55.62	52.42	47.97	39.12	<0.001	0.893
F pr. (Treatment)	<0.001	<0.001	<0.001	<0.001	<0.001		
l.s.d.	0.927	0.859	0.86	0.861	1.097		

**DBA Aurora (SC)**	5DPA	15DPA	25DPA	35DPA	45DPA	F pr. (DPA)	l.s.d.
CG	375.22	361.17	374.20	283.25	220.10	<0.001	32.21

WS	135.85	139.28	115.63	109.23	91.20	<0.001	15.46
HS	174.43	161.23	134.92	116.65	97.08	<0.001	16.94
WSHS	125.62	113.82	107.93	90.23	77.43	<0.001	14.24
F pr. (Treatment)	<0.001	<0.001	<0.001	<0.001	<0.001		
l.s.d.	22.69	21.79	21.16	21.89	18.66		

**DBA Spes (SC)**	5DPA	15DPA	25DPA	35DPA	45DPA	F pr. (DPA)	l.s.d.
CG	365.00	355.55	342.40	317.33	265.45	<0.001	32.26
WS	131.60	123.87	114.55	106.30	98.37	<0.001	18.53
HS	160.43	148.55	127.55	107.93	92.95	<0.001	17.17
WSHS	115.17	97.38	84.72	75.95	67.98	<0.001	14.01
F pr. (Treatment)	<0.001	<0.001	<0.001	<0.001	<0.001		
l.s.d.	12.44	11.22	10.17	9.77	8.56		

**EGA Bellaroi (SC)**	5DPA	15DPA	25DPA	35DPA	45DPA	F pr. (DPA)	l.s.d.
CG	321.05	331.70	308.50	267.02	195.98	<0.001	31.50
WS	74.40	71.32	63.73	53.75	43.67	<0.001	13.80
HS	95.20	74.97	64.47	67.32	46.42	<0.001	12.64
WSHS	66.03	58.28	59.47	47.68	42.45	<0.001	10.18
F pr. (Treatment)	<0.001	<0.001	<0.001	<0.001	<0.001		
l.s.d.	21.32	21.21	19.05	16.89	17.38		

**WID802 (SC)**	5DPA	15DPA	25DPA	35DPA	45DPA	F pr. (DPA)	l.s.d.
CG	310.28	324.05	308.73	273.17	210.97	<0.001	30.63
WS	93.98	89.38	79.40	69.62	57.58	<0.001	14.65
HS	126.75	106.97	89.05	74.87	60.82	<0.001	17.50
WSHS	85.82	77.25	71.93	61.68	52.67	<0.001	12.81
F pr. (Treatment)	<0.001	<0.001	<0.001	<0.001	<0.001		
l.s.d.	24.56	22.73	19.51	18.94	14.88		

**L1 (SC)**	5DPA	15DPA	25DPA	35DPA	45DPA	F pr. (DPA)	l.s.d.
CG	340.13	325.52	315.95	247.97	187.53	<0.001	28.72
WS	91.88	81.75	71.70	58.32	55.78	<0.001	10.78
HS	99.75	85.80	74.73	67.03	50.38	<0.001	10.36
WSHS	83.43	77.15	64.82	58.15	44.80	<0.001	8.37
F pr. (Treatment)	<0.001	<0.001	<0.001	<0.001	<0.001		
l.s.d.	8.9	9.04	8.82	7.28	6.17		

**L2 (SC)**	5DPA	15DPA	25DPA	35DPA	45DPA	F pr. (DPA)	l.s.d.
CG	377.75	385.58	378.55	317.68	275.70	<0.001	30.78
WS	157.80	150.47	156.85	130.67	109.05	<0.001	16.62
HS	151.13	144.02	132.48	121.40	98.07	<0.001	14.23
WSHS	142.25	135.67	124.22	108.95	89.12	<0.001	13.31
F pr. (Treatment)	<0.001	<0.001	<0.001	<0.001	<0.001		

l.s.d.	10.99	9.15	11.15	8.9	8.02		

**L3 (SC)**	5DPA	15DPA	25DPA	35DPA	45DPA	F pr. (DPA)	l.s.d.
CG	390.88	368.27	379.00	284.75	227.33	<0.001	32.16
WS	84.50	77.20	72.27	59.90	49.08	<0.001	9.79
HS	78.67	72.52	67.32	52.90	46.70	<0.001	8.89
WSHS	73.98	65.70	60.28	49.10	45.87	<0.001	8.01
F pr. (Treatment)	<0.001	<0.001	<0.001	<0.001	<0.001		
l.s.d.	9.25	9.34	8.71	8.22	7.68		

**L4 (SC)**	5DPA	15DPA	25DPA	35DPA	45DPA	F pr. (DPA)	l.s.d.
CG	347.72	340.88	356.57	284.90	233.20	<0.001	34.79
WS	103.63	95.40	84.25	77.07	72.85	<0.001	11.85
HS	123.82	111.02	99.08	72.38	61.20	<0.001	14.04
WSHS	88.72	82.30	73.40	65.82	60.33	<0.001	8.65
F pr. (Treatment)	<0.001	<0.001	<0.001	<0.001	<0.001		
l.s.d.	9.96	10.11	11.24	8.05	9.25		

**L5 (SC)**	5DPA	15DPA	25DPA	35DPA	45DPA	F pr. (DPA)	l.s.d.
CG	389.80	367.00	382.82	335.48	255.48	<0.001	37.78
WS	136.80	123.45	108.75	91.15	72.38	<0.001	17.00
HS	128.82	116.02	95.87	76.40	56.97	<0.001	14.68
WSHS	100.77	92.87	82.92	74.40	53.90	<0.001	10.72
F pr. (Treatment)	<0.001	<0.001	<0.001	<0.001	<0.001		
l.s.d.	11.76	11.53	11.13	10.67	9.71		

**L6 (SC)**	5DPA	15DPA	25DPA	35DPA	45DPA	F pr. (DPA)	l.s.d.
CG	356.88	367.83	354.20	263.25	235.10	<0.001	35.70
WS	86.52	81.28	73.08	57.57	51.20	<0.001	12.18
HS	114.43	88.23	76.92	54.17	44.92	<0.001	12.78
WSHS	72.28	64.48	60.43	51.75	41.60	<0.001	9.80
F pr. (Treatment)	<0.001	<0.001	<0.001	<0.001	<0.001		
l.s.d.	24.92	20.72	20.9	19.55	16.97		

**DBA Aurora (RWC)**	5DPA	15DPA	25DPA	35DPA	45DPA	F pr. (DPA)	l.s.d.
CG	95.23%	93.55%	91.84%	84.33%	72.93%	<0.001	2.28%
WS	93.32%	90.66%	87.34%	77.65%	66.80%	<0.001	2.66%
HS	91.98%	90.08%	86.15%	76.48%	64.69%	<0.001	2.66%
WSHS	91.23%	88.10%	84.46%	77.41%	63.93%	<0.001	2.69%
F pr. (Treatment)	0.057	0.014	0.002	<0.001	<0.001		
l.s.d.		2.83%	2.97%	1.89%	2.58%		

**DBA Spes (RWC)**	5DPA	15DPA	25DPA	35DPA	45DPA	F pr. (DPA)	l.s.d.

CG	94.71%	93.53%	93.05%	87.43%	80.65%	<0.001	2.44%
WS	92.30%	90.27%	84.75%	79.97%	71.72%	<0.001	2.23%
HS	92.52%	89.24%	85.58%	79.21%	71.01%	<0.001	2.17%
WSHS	90.75%	86.01%	81.44%	78.04%	68.07%	<0.001	2.50%
F pr. (Treatment)	0.004	<0.001	<0.001	<0.001	<0.001		
l.s.d.	1.64%	1.80%	2.25%	2.77%	3.27%		

**EGA Bellaroi (RWC)**	5DPA	15DPA	25DPA	35DPA	45DPA	F pr. (DPA)	l.s.d.
CG	95.10%	93.76%	93.21%	82.32%	74.79%	<0.001	2.87%
WS	87.40%	85.16%	80.81%	71.79%	64.27%	<0.001	3.16%
HS	91.41%	88.23%	83.10%	72.51%	64.92%	<0.001	2.76%
WSHS	85.43%	84.11%	79.72%	71.22%	63.97%	<0.001	2.66%
F pr. (Treatment)	<0.001	<0.001	<0.001	<0.001	<0.001		
l.s.d.	2.07%	2.80%	3.89%	2.57%	3.19%		

**WID802 (RWC)**	5DPA	15DPA	25DPA	35DPA	45DPA	F pr. (DPA)	l.s.d.
CG	94.65%	92.82%	89.74%	86.76%	75.88%	<0.001	2.78%
WS	89.30%	87.10%	84.38%	80.40%	68.30%	<0.001	2.68%
HS	91.37%	89.86%	85.07%	80.22%	67.64%	<0.001	2.52%
WSHS	87.54%	84.27%	80.96%	76.72%	65.69%	<0.001	2.97%
F pr. (Treatment)	<0.001	<0.001	<0.001	<0.001	0.001		
l.s.d.	2.14%	2.57%	2.95%	2.45%	3.80%		

**L1 (RWC)**	5DPA	15DPA	25DPA	35DPA	45DPA	F pr. (DPA)	l.s.d.
CG	94.22%	92.82%	90.64%	85.53%	67.36%	<0.001	2.32%
WS	87.77%	81.74%	79.39%	76.51%	58.40%	<0.001	2.98%
HS	88.02%	85.31%	82.95%	77.74%	60.30%	<0.001	1.99%
WSHS	85.58%	79.90%	77.80%	73.74%	54.50%	<0.001	4.03%
F pr. (Treatment)	<0.001	<0.001	<0.001	<0.001	0.001		
l.s.d.	2.75%	2.92%	2.30%	2.37%	3.80%		

**L2 (RWC)**	5DPA	15DPA	25DPA	35DPA	45DPA	F pr. (DPA)	l.s.d.
CG	95.04%	94.36%	90.41%	85.81%	76.78%	<0.001	1.84%
WS	93.52%	91.26%	85.74%	79.61%	69.03%	<0.001	2.22%
HS	92.57%	89.13%	84.12%	78.54%	68.02%	<0.001	2.28%
WSHS	90.17%	88.41%	83.61%	76.04%	65.92%	<0.001	2.57%
F pr. (Treatment)	0.002	<0.001	<0.001	<0.001	<0.001		
l.s.d.	1.79%	1.98%	2.48%	2.68%	2.54%		

**L3 (RWC)**	5DPA	15DPA	25DPA	35DPA	45DPA	F pr. (DPA)	l.s.d.
CG	95.19%	92.29%	90.64%	87.29%	72.93%	<0.001	2.58%
WS	89.30%	84.96%	82.02%	73.74%	61.77%	<0.001	2.60%
HS	91.63%	84.14%	82.15%	74.54%	60.49%	<0.001	2.32%

WSHS	88.72%	83.01%	77.49%	70.06%	55.42%	<0.001	2.47%
F pr. (Treatment)	0.001	<0.001	<0.001	<0.001	<0.001		
l.s.d.	2.46%	2.21%	2.39%	2.71%	3.05%		

**L4 (RWC)**	5DPA	15DPA	25DPA	35DPA	45DPA	F pr. (DPA)	l.s.d.
CG	94.37%	93.49%	91.00%	85.89%	75.55%	<0.001	2.56%
WS	88.67%	86.23%	82.69%	73.15%	62.50%	<0.001	2.25%
HS	87.85%	85.32%	82.52%	72.46%	60.49%	<0.001	2.22%
WSHS	85.16%	83.44%	79.57%	72.28%	56.77%	<0.001	2.12%
F pr. (Treatment)	0.001	<0.001	<0.001	<0.001	<0.001		
l.s.d.	1.83%	2.13%	1.99%	1.99%	3.52%		

**L5 (RWC)**	5DPA	15DPA	25DPA	35DPA	45DPA	F pr. (DPA)	l.s.d.
CG	95.12%	93.33%	90.30%	86.99%	81.89%	<0.001	1.83%
WS	90.95%	85.16%	81.81%	76.43%	71.11%	<0.001	2.31%
HS	92.48%	86.16%	78.81%	73.05%	67.72%	<0.001	2.51%
WSHS	90.67%	85.28%	78.33%	72.17%	63.88%	<0.001	2.38%
F pr. (Treatment)	0.003	<0.001	<0.001	<0.001	<0.001		
l.s.d.	2.02%	1.86%	2.29%	2.19%	3.17%		

**L6 (RWC)**	5DPA	15DPA	25DPA	35DPA	45DPA	F pr. (DPA)	l.s.d.
CG	95.23%	93.11%	92.29%	83.59%	73.73%	<0.001	1.92%
WS	89.85%	84.81%	80.41%	71.07%	59.96%	<0.001	2.74%
HS	91.70%	86.99%	83.47%	71.93%	60.74%	<0.001	2.62%
WSHS	85.84%	82.35%	79.10%	70.03%	57.46%	<0.001	2.23%
F pr. (Treatment)	<0.001	<0.001	<0.001	<0.001	<0.001		
l.s.d.	2.74%	1.95%	2.17%	2.29%	3.10%		

Supplementary Figure S1. A schematic representation of different stress treatments applied in this study. CG, control group; WS, water stress group; HS, heat stress group; WSHS, combined water stress and heat stress group. 22/12°C and 37/27°C represent day/night temperatures with a 12h photoperiod. Field capacity was 12% soil water content.

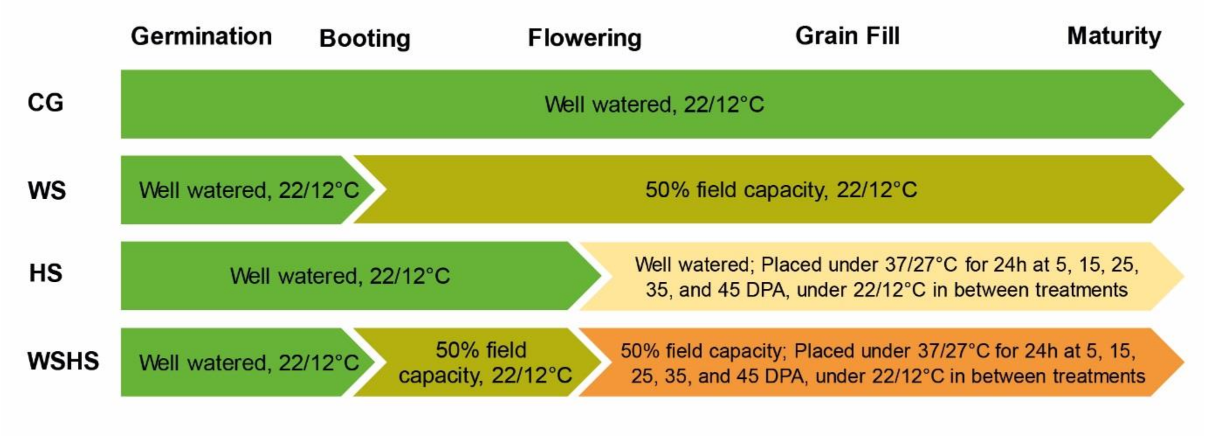

Supplementary Figure S2. Images of stress-sensitive genotype L6 at five time-points in four treatment groups. CG, control group; WS, water stress group; HS, heat stress group; WSHS, combined water stress and heat stress group. The pot size was 8.5 cm × 8.5 cm × 18 cm.

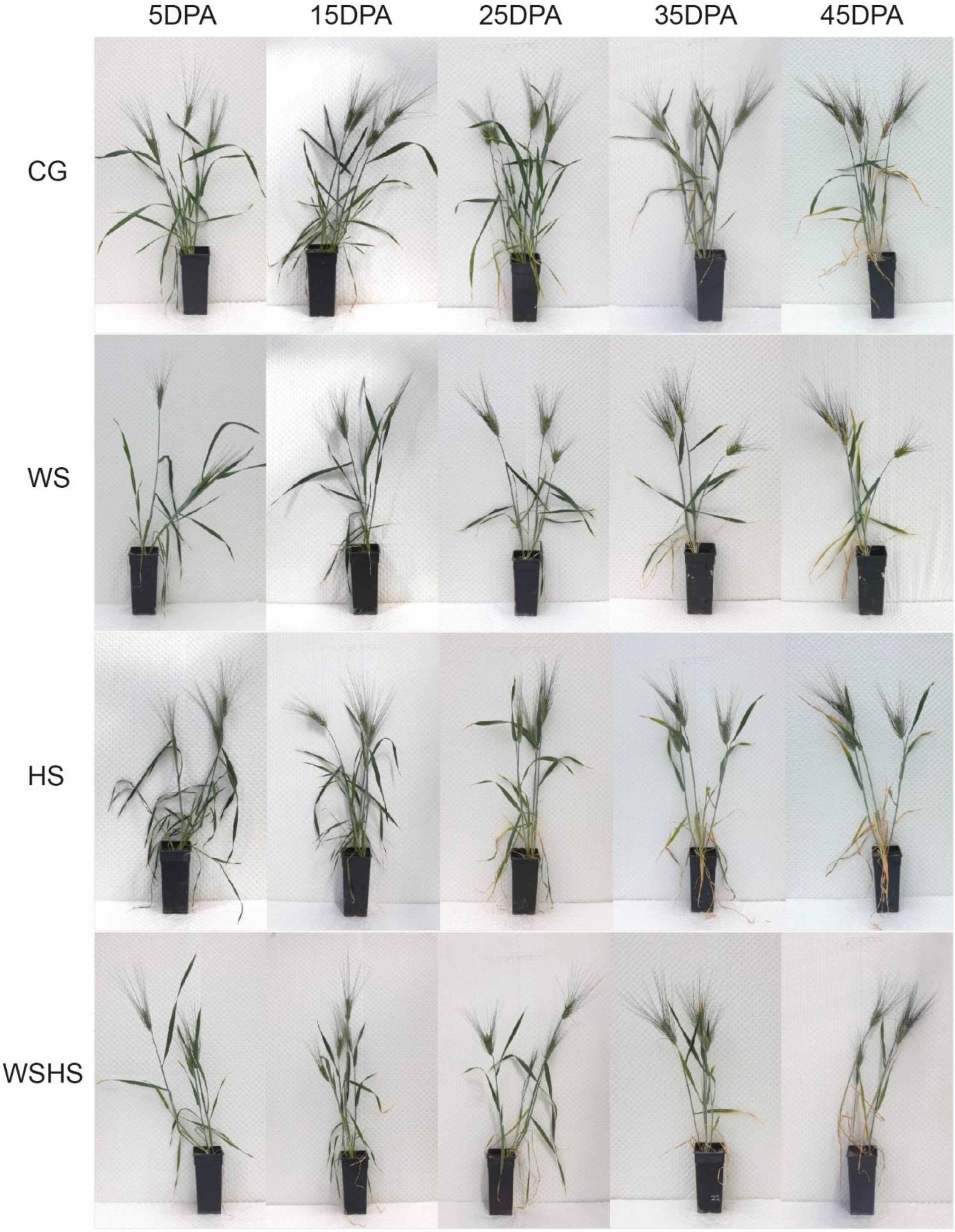

Supplementary Figure S3. Images of stress-tolerant DBA Aurora at five time-points in four treatment groups. CG, control group; WS, water stress group; HS, heat stress group; WSHS, combined water stress and heat stress group. The pot size was 8.5 cm × 8.5 cm × 18 cm.

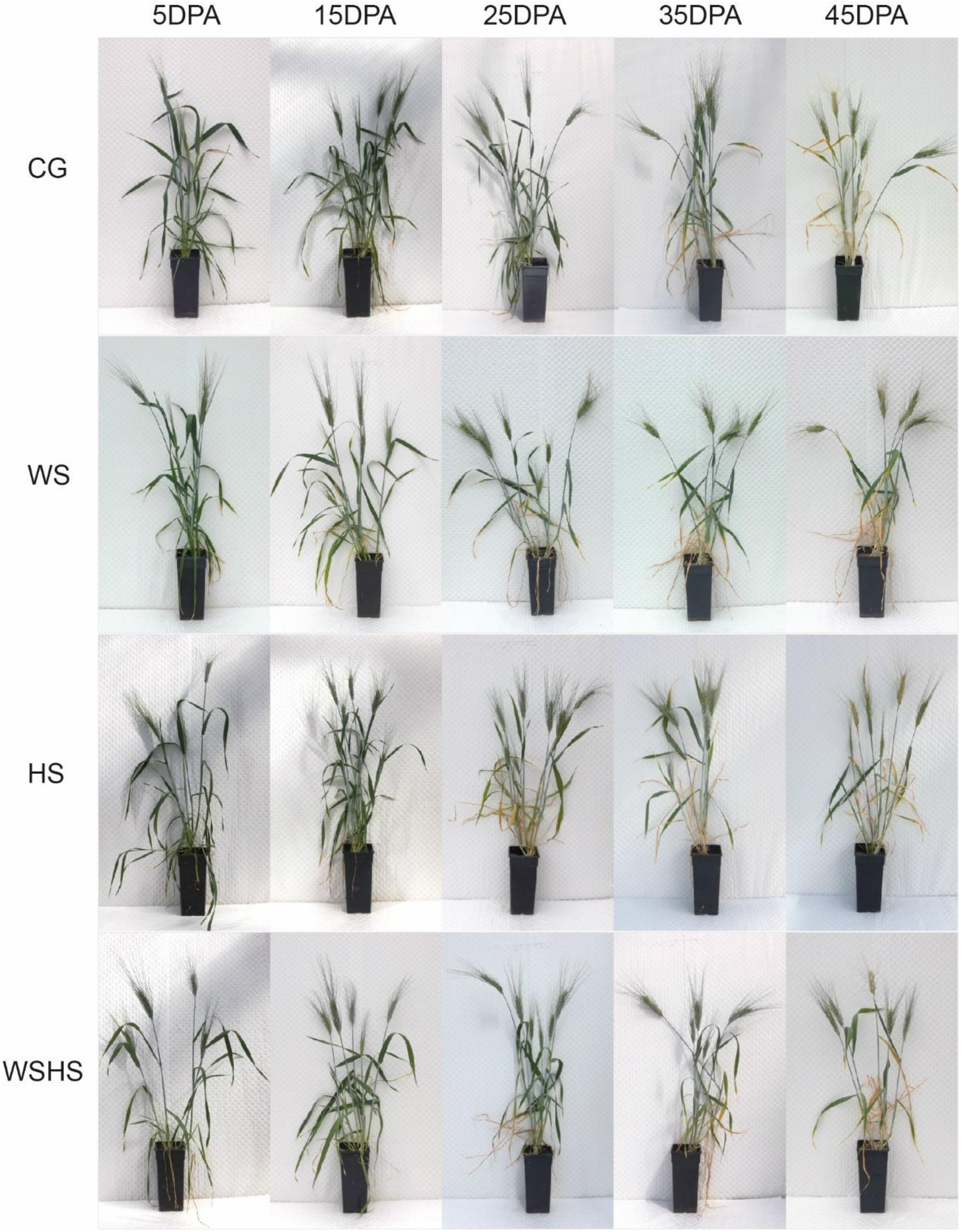

Supplementary Figure S4. Weight of all spikes per plant **(a)**, spike harvest index **(b)**, spikelet number per plant **(c)**, grain number per spike **(d)**, and spikelet number per spike **(e)** of ten durum wheat genotypes in four treatment groups. Means ± SE for $n = 6$ are shown. Different letters (a, b, c) denote statistically significant differences at the $P < 0.05$ level among treatment groups. When no letters were shown there was no statistical difference ($P > 0.05$) detected among the treatment groups. CG, control group; WS, water stress group; HS, heat stress group; WSHS, combined water stress and heat stress group.

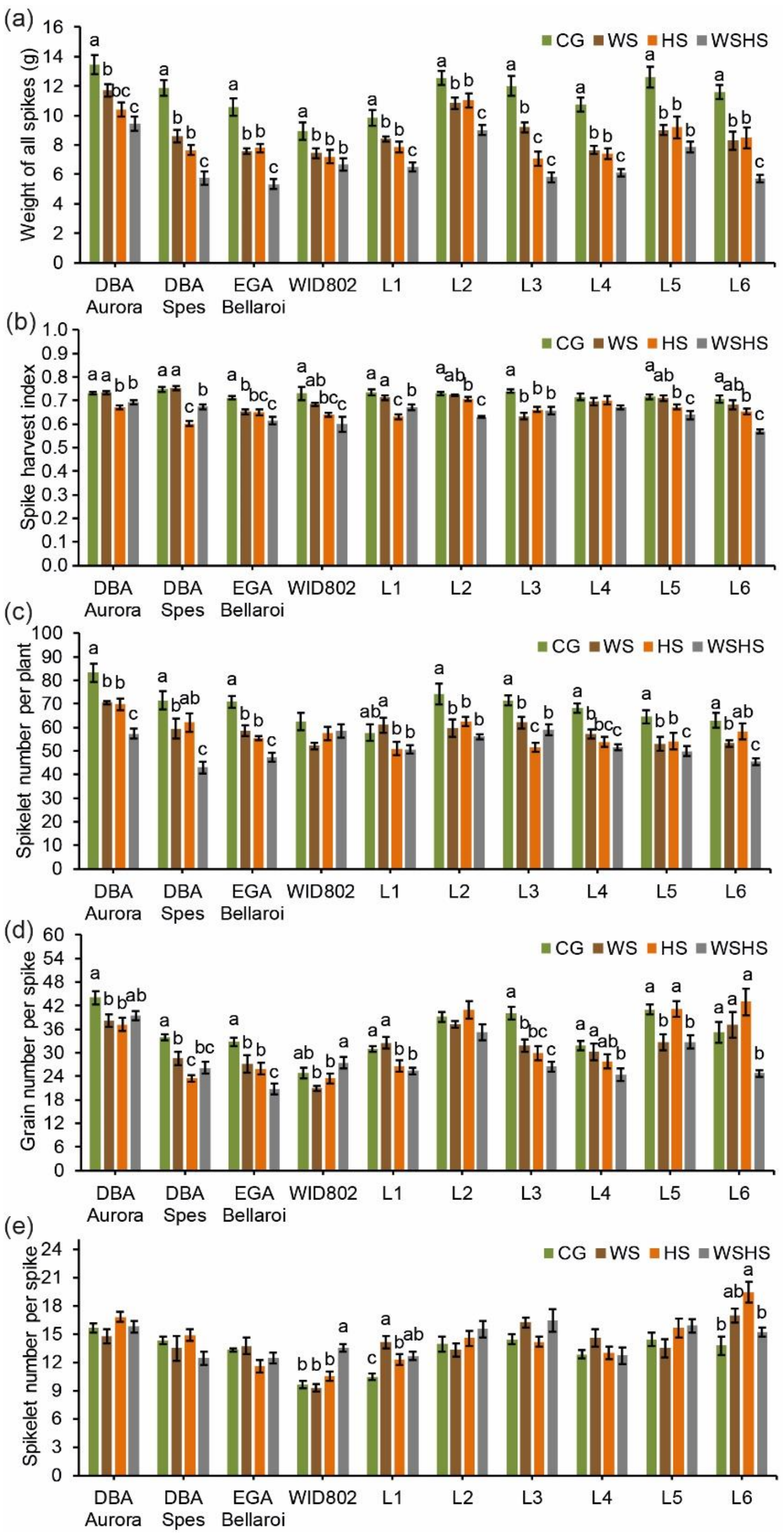

Supplement: Supplementary file 1 — Supplementary information [file 41598_2019_49871_MOESM1_ESM.pdf]
